# Human metapneumovirus epidemiological and evolutionary patterns in Coastal Kenya, 2007-11

**DOI:** 10.1186/s12879-016-1605-0

**Published:** 2016-06-17

**Authors:** Betty E. Owor, Geoffrey N. Masankwa, Lilian C. Mwango, Regina W. Njeru, Charles N. Agoti, D. James Nokes

**Affiliations:** Kenya Medical Research Institute (KEMRI) -Wellcome Trust Research Programme, Kilifi, KEMRI Centre for Geographic Medicine Research - Coast, Kilifi, Kenya; Department of Biomedical Sciences, Pwani University, Kilifi, Kenya; School of Life Sciences and WIDER, University of Warwick, Coventry, UK

**Keywords:** Human metapneumovirus, Genetic diversity, Respiratory virus, Kenya, Kilifi

## Abstract

**Background:**

Human metapneumovirus (HMPV) is an important global cause of severe acute respiratory infections in young children and the elderly. The epidemiology of HMPV in sub-Saharan Africa is poorly described and factors that allow its recurrent epidemics in communities not understood.

**Methods:**

We undertook paediatric inpatient surveillance for HMPV in Kilifi County Hospital (KCH) of Coastal Kenya between 2007 and 2011. Nasopharyngeal samples collected from children aged 1 day–59 months admitted with severe or very severe pneumonia, were tested for HMPV using real-time polymerase chain reaction (RT-PCR). Partial nucleotide sequences of the attachment (G) and fusion (F) surface proteins of positive samples were determined and phylogenetically analyzed.

**Results:**

HMPV was detected in 4.8 % (160/3320) of children [73.8 % (118/160) of these less than one year of age], ranging between 2.9 and 8.8 % each year over the 5 years of study. HMPV infections were seasonal in occurrence, with cases predominant in the months of November through April. These months frequently coincided with low rainfall, high temperature and low relative humidity in the location. Phylogenetic analysis of partial F and G sequences revealed three subgroups of HMPV, A2 (74 %, 91/123), B1 (3.2 %, 4/123) and B2 (22.8 %, 28/123) in circulation, with subgroup A2 predominant in majority of the epidemic seasons. Comparison of G sequences (local and global) provided a greater phylogenetic resolution over comparison of F sequences and indicated presence of probable multiple G antigenic variants within the subgroups due to differences in amino acid sequence, encoded protein length and glycosylation patterns.

**Conclusion:**

The present study reveals HMPV is an important seasonal contributor to respiratory disease hospitalization in coastal Kenya, with an evolutionary pattern closely relating to that of respiratory syncytial virus.

**Electronic supplementary material:**

The online version of this article (doi:10.1186/s12879-016-1605-0) contains supplementary material, which is available to authorized users.

## Background

Human metapneumovirus (HMPV), a close relative of respiratory syncytial virus (RSV), is a recognized major human pathogen that causes epidemics of respiratory tract illnesses in persons of all ages worldwide [1, 2]. Discovered in 2001 [1], HMPV was probably circulating for at least 50 years prior to this date [1]. Infection with HMPV may manifest as upper or lower tract respiratory illness, similar to that observed with RSV disease although HMPV has a considerably lower individual disease risk and population burden than RSV [3, 4].

A member of the *Paramyxoviridae* family of viruses, HMPV genome is a negative-sense single-stranded RNA molecule, 13.3 Kb long, encoding eight proteins [5]. Three surface proteins F (fusion), G (attachment glycoprotein) and SH (small hydrophobic) are encoded within the HMPV genome [6] and F and G nucleotide sequences have been largely used to study HMPV genetic variation [7]. Whilst the G gene shows higher sequence and amino acid diversity [8–11], only the F protein is confirmed to be immunogenic and protective [6, 12].

In the northern hemisphere peak HMPV disease occurrence is typically in winter and spring months of January to May [13–15], while in the southern hemisphere peak prevalence is in the spring period of August to September [16]. In Kenya, peak HMPV prevalence has been recorded in June-July in the west and November-December in refugee camps in the northeast and northwest of the country [17, 18].

Worldwide, HMPV prevalence in hospital inpatient or community studies, in children or elderly adults, varies widely from as low as 1.7 % to as high as 17 %, with generally higher prevalence in outpatients compared to inpatients and, also, more in children younger than 5 years compared to older age groups [7, 13, 14, 16, 19–26]. Studies in Kenya report HMPV prevalence between 3 and 6 % in acute respiratory infection cases in inpatient populations [17, 27–29], and 7 to 8.6 % in outpatient [17, 30] but none have provided information on virus genetic characteristics and underlying evolutionary changes over successive epidemic seasons.

HMPV has been divided into two serologically distinct groups, A and B [1, 31]. Group A generally dominates over group B [7, 24–26, 32] and has been reported to cause more severe disease than group B [33]. The two groups are further subdivided into subgroups A1, A2, B1 and B2 based on genetic differences in the surface proteins F and G but these do not show clear antigenic differences at least in neutralization assays using anti-sera raised in ferrets [8]. The A2 subgroup is the most genetically heterogeneous of the four subgroups and some studies have suggested its further sub-division into A2a and A2b sub-lineages based on sequence data [34, 35]. Based on the F gene, HMPV groups A and B have 84–86 % homology at nucleotide level and 94–97 % at amino acid level whilst within subgroup similarity is 94–96 % at nucleotide and 97–99 % at amino acid levels [8]. In comparison, the more diverse G protein shows only 50–57 % and 30–37 % similarity for nucleotide and amino acid sequences, respectively, between the two groups A and B [8]. Variants from both groups, and sometimes from multiple subgroups within the groups, can co-circulate in the same epidemic season [8, 12, 14, 35, 36].

Candidate vaccines targeting the G protein and a subunit vaccine of the F protein have shown promising results although, to date, none is licensed [37–40]. We set out to understand the genetic diversity in the F and G genes in circulating strains in coastal Kenya in relation to seasonal introductions of the virus, to contribute information that may be important for vaccine development and virus infection control. We describe the molecular epidemiology of HMPV in child admissions at a coastal county hospital of Kenya, over a 5-year period, building on previous work in the hospital [27] in order to elucidate prevalence, circulating strains and genetic diversity in the most at risk paediatric population, contributing information on HMPV persistence and transmission.

## Methods

### Study population and sample collection

Study participants were identified through continuous surveillance of pneumonia admissions to the paediatric wards of Kilifi County Hospital (KCH) over a 5-year period between January 2007 and December 2011. KCH is located in coastal Kenya, 60 km north of Mombasa and is the main hospital that serves the residents of Kilifi County. The hospital handles 4000 to 4500 paediatric admissions annually and around 30 % of the under 5 year olds have an admission diagnosis of lower respiratory tract infection (LRTI) based on WHO definitions of severe and very severe pneumonia [41]. Hospital admissions can be further stratified according to residency within the Kilifi Health and Demographic Surveillance System, KHDSS [42]. Each year this coastal location experiences two rainy seasons: long rains between April and July and short rains between October and December. Further details of the location and ongoing surveillance have previously been described [27, 28, 43, 44].

Children were eligible for the current study if at admission they were aged 1 day to 59 months with syndromic severe or very severe pneumonia, i.e. cough or difficulty in breathing plus any one or more of the following: lower chest wall indrawing (severe pneumonia), oxygen saturation of less than 90 % (finger tip pulse oximetry), inability to drink or breast feed, prostrate or unconscious (very severe pneumonia) [27]. Following written informed consent from the parent or guardian, a nasopharyngeal flocked swab, nasal wash or combination of nasopharyngeal swab and oropharyngeal swab was collected from each child, placed in 3 ml viral transport medium, and stored at −80 °C prior to laboratory screening. The Kenya National Ethical Review Committee approved the study protocols.

### Diagnostic real-time polymerase chain reaction (RT-PCR)

RNA was extracted from either 200 or 140 μl of nasopharyngeal samples using MagNA Pure LC32 automated total nucleic acid extractor (Roche Applied Science, Mannheim, Germany) or QIAamp Viral RNA minikit (Qiagen, Valencia, CA, USA), respectively, according to the manufacturer’s instructions, for virus screening and sequencing respectively. Virus detection was done using real-time PCR using a TaqMan probe based system as described by Hammitt et al., [28] in a multiplex PCR assay run on the ABI 7500 (version 2.5, Applied Biosystems, Foster City, California, USA). Samples with a cycle threshold (Ct) values of less than 35.0 were considered positive and taken through to sequencing. Samples from 2007 were processed as described in Berkley et al., [27]. In this case, RNA was extracted from 200 ul of nasal sample using the Magnapure LC Total Nucleic Acid Isolation Kit (Roche, Manheim, Germany) and virus detection conducted using the LightCycler Fast Start DNA MasterPLUS Hyb-Probe kit (Roche, Mannheim, Germany).

### Gene specific PCR and sequencing

Surface proteins encoding genes, the fusion protein (F) and glycoprotein (G), were amplified in a one-step RT-PCR assay using Quantifast one-step RT-PCR system (Qiagen, Valencia, CA, USA). Primers targeting the ectodomain region of the F protein to give a 405 bp product were used to amplify a portion of the F gene as previously described [45]. Amplification of the G gene was performed using semi-nested PCR to yield a 930 bp product that included the G gene and a portion of the L gene [7]. Amplified products of both the F and G gene were checked on a 2 % agarose gel with Ethidium Bromide staining to ascertain successful amplification. The remainder of the PCR products were purified using GFX DNA purification kit (GFX-Amersham, Amersham, UK) according to the manufacturer’s instructions and taken forward for DNA sequencing. Because of PCR failures at the amplification stage, only 130 and 98 samples (out of the total of 160) were followed through to sequencing for F and G gene, respectively. Purified PCR products were sequenced using Big Dye Terminator 3.1 (Applied Biosystems, Foster City, California, USA) using the same PCR primers in both forward and reverse direction and generated with an ABI Prism 3130xl Genetic Analyzer (Applied Biosystems, Foster City, California, USA).

### Sequence alignment, phylogenetic and molecular analysis

Raw sequences were assembled using either DNASTAR or Sequencher (version 4.10.1, Gene Codes Corporation, Ann Arbor, USA). Multiple sequence alignments (MSA) were undertaken in MAFFT v7.220 [46]. G gene sequences were trimmed to approximately 606 bp to remove the intergenic region and a portion of the L gene. To obtain comparison data, a GenBank search was conducted on 11-Jun-2015. The search terms were “human Metapneumovirus AND (F OR Fusion) AND 340[SLEN]:14000[SLEN]” for F protein data, and “human metapneumovirus attachment AND 600[SLEN]:14000[SLEN]” for G protein data. To be included in our comparison dataset the sequences had to have a complete overlap in their sequenced portion with Kilifi virus data, information on the country sampled and sampling date (at least year) of between 2007 and 2011. With these criteria we identified 290 (for F) and 233 (for G) sequences. Duplicate sequences were dropped. Genbank accession numbers of sequences for these analyses are available in Additional file 1: Table S1 for F gene and Table S2 for G gene.

Phylogenetic trees were generated using both Maximum Likelihood (ML) and Bayesian methods: For ML in MEGA v5.2.2 and for Bayesian in BEAST (Bayesian evolutionary analysis and sampling of Trees) v1.8.2. To genotype the Kilifi viruses, the F protein sequence data were analyzed with reference sequences deposited in GenBank (details provided in Additional file 1: Table S1). A genotype was only confirmed if sequences clustered with the reference sequences within a major branch with >70 % bootstrap support on the ML tree. Temporally structured phylogenetic trees were generated using BEAST. Tip dates (dd-MMM-yyyy) were used in all analyses. For comparison dataset sequences that had only the year of collection, date of collection was estimated to 01st of July of the reported year. All Bayesian analyses used HKY, gamma distribution with invariant sites as the model of evolution and demographic model of constant population size. The analysis was set to 50 million steps sampling after every 2500 steps. The output was only further analyzed when ESS (estimated sample size) for all parameters exceeded 200. We settled on the above parameters in the Bayesian analyses after alternative more complex models e.g. general-time reversible (GTR) failed to give a converged result. Maximum clade credibility trees were calculated using Tree Annotator 1.8.2. and visualized in Fig Tree v1.4.2.

To analyze variation in sequences, unique sequences and variable nucleotide and amino acid positions were identified using in-house python and ruby scripts. Patristic distances were analyzed in MEGA v5.2.2 [47]. Sequences generated in this study are deposited in GenBank under the accession numbers: KT191355-KT191484 for F and KT191299-KT191354 for G protein.

## Results

### Study population

Between January 2007 and December 2011, there were 16,439 admissions to KCH aged between 1 day and 59 months, of which 32.1 % (5284) were eligible for study as cases with syndromic severe or very severe pneumonia (Table 1). Overall, 62.8 % of these children were tested for HMPV, ranging by year between 43 and 83 % due to changes in proportion of non-residents of KHDSS included in the different samples (15.4 % in years 2007–09, versus 47.3 % in years 2010–11).

**Table 1:** Study population at Kilifi County Hospital, number tested and number of HMPV positive samples recorded

	Number of children			HMPV positive samples and genotypes				
Year	Admissions	Eligible	Tested (%)	Number positive (%) ^a^	Genotyped (%) ^b^	A2	B1	B2
2007	3506	1174	726 (61.8)	21 (2.89)	10 (48)	4	2	4
2008	3204	1009	435 (43.1)	34 (7.82)	29 (85)	21	2	6
2009	3633	1105	526 (47.6)	46 (8.75)	35 (76)	26	0	9
2010	3091	1090	903 (82.8)	27 (2.99)	23 (85)	20	0	3
2011	3005	906	730 (80.6)	32 (4.38)	26 (81)	20	0	6
Total	16,439	5284	3320 (62.8)	160 (4.82)	123 (77)	91	4	28

^a^% number positive/tested

^b^% genotyped/number positive

### HMPV prevalence in child admissions

HMPV was detected in 160 (4.8 %) of the 3320 samples tested. Prevalence by year ranged from 2.9 % in 2007 to 8.8 % in 2009 (Table 1). Almost half of HMPV positive samples were identified in the years 2008 and 2009. Children under 6 months of age accounted for 44 % of cases (71/160) while 74 % (118/160) of cases were in children under 1 year old with only 1.3 % (2/160) HMPV positive for children >36 months (Table 2). Of the 160 HMPV positive cases, 83.8 % (134/160) and 16.2 % (26/160) presented with symptoms classified as either severe or very severe pneumonia, respectively (Table 2).

**Table 2:** HMPV positives stratified by age group of patients and pneumonia status in Kilifi County Hospital

Age group (months)	Eligible	Tested	HMPV positive (%) ^a^	Genotype		
				A2 (%) ^b^	B1	B2
0–2	1479	901	29 (18.1)	12 (9.8)	2	5
3–5	778	524	42 (26.3)	24 (19.5)	1	6
6–11	1143	703	47 (29.4)	32 (26.0)	0	5
12–23	1056	682	25 (15.6)	12 (9.8)	1	9
24–35	440	269	15 (9.4)	10 (8.1)	0	2
36+	388	241	2 (1.3)	1 (0.81)	0	1
Total	5284	3320	160 (4.82)	91 (74)	4	28
Admission condition						
Severe pneumonia	3783	2482	134	73	4	23
Very severe pneumonia	1501	838	26	18	0	5
All pneumonia (severe and very severe)	5284	3320	160	91	4	28

^a^% HMPV positive/total HMPV positive; ^b^( ) proportion of sequenced samples (genotype/total genotyped)

### Temporal occurrence and circulation patterns of HMPV

**Fig. 1:**
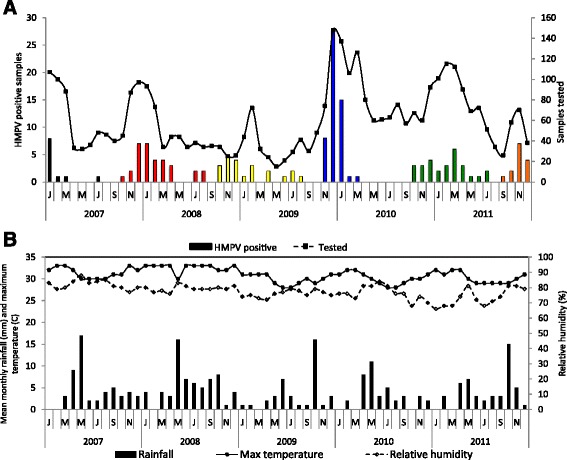
**a** Temporal distribution of HMPV positive samples in Kilifi over five years, showing number of positive samples each month on the primary axis and number of samples tested monthly on the secondary axis. Different *colours* indicate the different epidemics the samples were assigned; **b** Monthly weather patterns in Kilifi, Kenya in the period 2007–2011

HMPV occurrence showed a seasonal pattern with the majority of cases being detected in the period from October of 1 year through to April of the next (Fig. 1a). The seasonal increase in cases tended to coincide with lower rainfall, higher temperature and lower relative humidity (Fig. 1b). For subsequent analysis we assume August as the end month of one season and September the start of the next (hence the colour scheme in Fig. 1a). However, there was no clear-cut demarcation between the end of one seasonal epidemic and the next as sporadic HMPV cases were detected in seasonal troughs, except for the inter-epidemic period between the end of the 2009-10 and rise of the 2010-11 seasons, where no cases were observed over a 6 month interval (Fig. 1a).

### Genetic diversity of HMPV samples from KCH

PCR amplification of the F gene was more successful than for the G gene, with 130 and 98 positive PCRs for F and G gene, respectively. A total of 123 samples from the 160 HMPV positives were successfully sequenced for both or either G or F gene only and genotyped (Table 1). There was no statistically significant difference (*P* = 0.613) in Ct values between sequenced samples and those that failed to be sequenced (numbering 37).

**Fig. 2:**
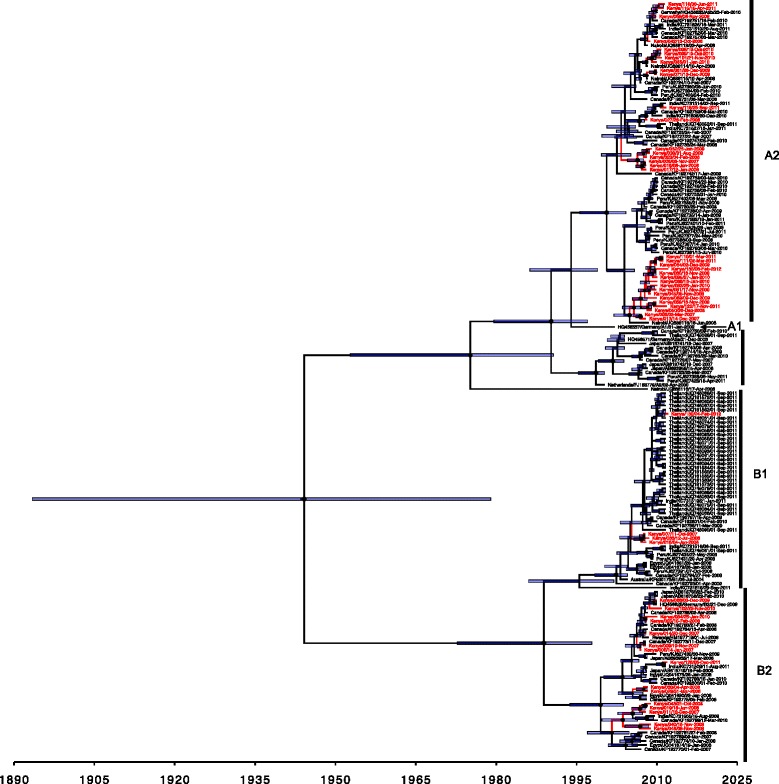
Phylogenetic relatedness and temporal divergence of the combined Kilifi and contemporaneous global F protein sequences over the 345-nucleotide portion analyzed. Taxa of Kilifi viruses are coloured *red. Node bars* indicate the 95 % HPD height interval of the nodes; the node makers size are scaled by posterior support, for Kilifi, coastal Kenya 2007–11

Among the 123 samples successfully sequenced for the F protein over a 345 nucleotide length region, 49 of these were unique. Overall mean nucleotide diversity for this subset was 0.106. In the phylogenetic analysis we combined the Kilifi unique F sequences with all others deposited in Genbank that were contemporaneous and overlapping in the sequence F portion. Both A and B HMPV groups, specifically A2, B1 and B2 were observed in Kilifi (Fig. 2). Subgroup A1 was not observed in Kilifi (Fig. 2). Within the subgroups, virus sequences from the same epidemic did not necessarily group together into marked clusters instead they were interspersed on the phylogenetic tree with the international sequences (Fig. 2). Majority of Kilifi sequences in the A2 subgroup occurred within three distinguishable clusters and when compared to global sequences, clustered closely with sequences from Canada and Nairobi Kenya and were highly similar in each of the subgroups in which they fell (Fig. 2). A ML phylogeny of the HMPV F sequences from Kilifi alone, color coded by epidemic is given in Additional file 2: Figure S1A. Notably, phylogenetic clusters formed within the different subgroups on this tree had sequences from multiple epidemic periods i.e. no clear temporal clustering.

Of the 98 PCR positives for the G protein, 56 samples sequenced successfully over the 606 nucleotides of the HMPV G coding region. This represented coverage of 88.2 % of the entire G coding sequence. All the 56 sequences were determined to be of genotype A2 within group A (figure not shown) with 53 providing unique sequences over the sequenced region. This unique subset showed an overall mean genetic diversity of 0.079. An ML phylogeny of the HMPV G sequences from Kilifi alone, color coded by epidemic is given in Additional file 2: Figure S1B.

The phylogenetic resolution was far greater with G sequences compared to F sequences (Additional file 2: Figure S1A), showing higher bootstrap support values and longer branch lengths. Viruses deemed identical in the F portion we sequenced possessed multiple nucleotide differences in the G portion (Additional file 2: Figure S1B). However, similar to what was observed in the F-based phylogeny, Kilifi sequences did not cluster strictly according to epidemic, but rather sequences from multiple epidemic periods frequently occurred within the phylogenetic clusters but these tended to be those deriving from successive epidemics (Additional file 2: Figure S1B).

**Fig. 3:**
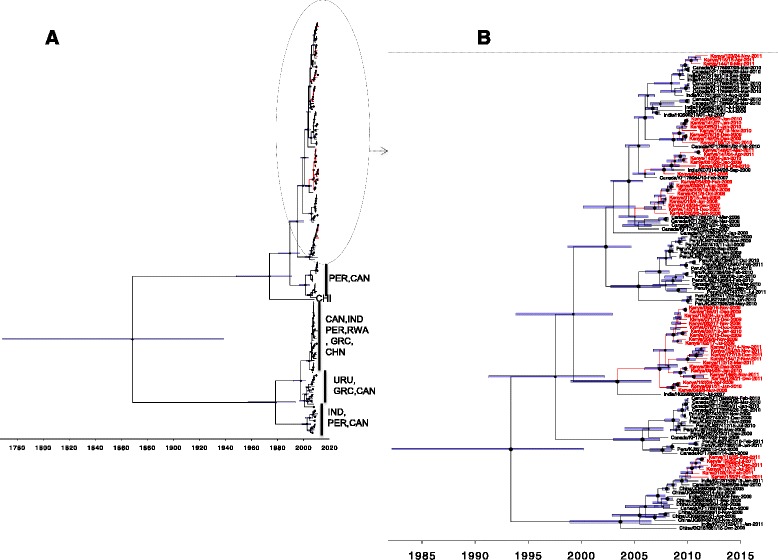
Phylogenetic and temporal placement of Kilifi group A G protein sequenced viruses, for Kilifi, coastal Kenya 2007–2011. Panel **a** A total of 209 viruses compared in G sequences G (53 from Kilifi and 156 collated from GenBank from 7 countries). Branches leading to Kilifi viruses are coloured *red*. Three letter codes of countries comprising branches without Kilifi representative sequences are indicated next to the *vertical line*. Panel **b** 121 viruses that fell within the ancestral node leading to Kilifi viruses were reanalyzed in BEAST. Again branches and leaves of Kilifi viruses are colored *red* on the phylogenetic temporally calibrated tree. *Node bars* indicate the 95 % HPD height interval of the nodes; the node maker sizes are scaled by posterior support. The number *1*, *2*, *3* and *4* represent the three major and one minor cluster of sequences from Kilifi

Comparison of Kilifi G gene sequences with global sequences showed that Kilifi sequences clustered closely with some sequences from Canada, Peru, China and India. However, there were clusters of sequences from Peru, Canada, India, Greece, Uruguay and Rwanda for which close relatives were absent in Kilifi (Fig. 3a). The Kilifi G sequences diverged into three major clusters (cluster 1, 2, 3 in Fig 3; Additional file 2: Figure S1B) and one minor cluster (4 in Fig. 3). Each cluster consisted of sequences from viruses from more than one epidemic; cluster 1 of epidemic 2010-11; cluster 2 of epidemics 2008-09, 2009-10 and 2011-12 and cluster 3 of epidemics 2007-08, 2008-09, 2009-10 and 2010-11. Within each cluster, sequences from the same epidemic grouped together. While cluster 1 was distinctly removed from the other clusters (Fig. 3) and majority of global sequences, it was closely related to sequences mainly from Asia specifically China and India. The major cluster of Kilifi sequences (cluster 2) consisting of 22 sequences was most closely related to one sequence from India. Sequences in cluster 3 were closely related to sequences mainly from Canada and a few sequences from India (Fig. 3). There was a unique branch of sequences mainly from Peru and one from China into which none of the Kilifi sequences fitted.

A temporal analysis of genotype occurrence and circulation in Kilifi showed that the majority (91/123) of circulating isolates were A2 and this type was dominant and circulating in each of the five epidemics (Additional file 3: Figure S2) while B1 (3.3 %, 4/123) and B2 (22.8 %, 28/123) occurred less frequently (Table 1; Additional file 3: Figure S2). Whereas A2 and B2 were recorded in every epidemic and two subgroups circulated concurrently in each epidemic, B1 was only present in epidemic 2007–2008 (Additional file 3: Figure S2).

### Subgroup prevalence patterns in Kilifi versus global

**Fig. 4:**
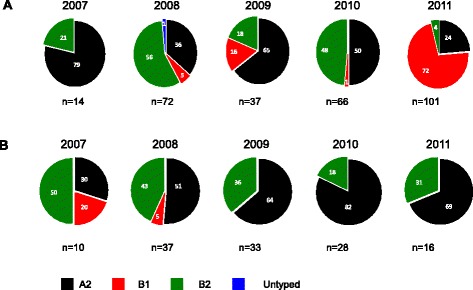
Pie charts showing the genotype distribution by year derived from F sequence analysis of samples from Kilifi, Coastal Kenya 2007-11. Panel **a** This is based on the 290 F sequences collated from GenBank. Panel **b** This is based on 123 F sequences generated from samples that were collected in this study at the KCH between 2007 and 2011. The *numbers* inside the pies indicate the genotype proportions per the respective year

We compared the subgroup prevalence in the 123 F sequences from Kilifi with 290 global sequences we collated from GenBank to show genotype distribution by year. The global dataset was drawn from seven countries: Japan, Peru, Rwanda, Egypt, Thailand, India and Canada. The patterns in Kilifi appeared considerably distinct from the overall global patterns (Fig. 4). Only the year 2010 in Kilifi mirrored genotypes trends that were observed globally, with subgroup A2 dominating (Fig. 4b).

### Evolutionary analysis

We estimated the overall evolutionary rate for the F region analysed from the combined Kilifi-global sequence dataset. It was determined as 1.96 × 10^−3^ substitutions/site/year (95 % HPD Interval: 1.37 × 10^−3^, 2.57 × 10^−3^). This is including all group A and B strains. Divergence dates of the groups A-B, subgroups A1-A2, and subgroup B1-B2 from these F data were estimated as, 1944.16 [95 % HPD interval 1893.4, 1979.0], 1994.0 [95 % HPD interval 1986.3, 1998.9] and 1988.9 [95 % HPD interval 1972.8, 1997.8], respectively (Fig. 2). A similar analysis determined the evolutionary rate in the G region we sequenced for the A2 genotype to be 5.915 × 10^−3^ substitutions/site/year (95 % HPD Interval: 4.147 × 10^−3^, 7.887 × 10^−3^).

### Analysis of protein changes in the F and G genes

The HMPV G protein is on average 236 amino acids long. For the Kilifi genotype A2 G protein sequences were predicted to encode 3 different protein lengths: 213, 217 or 228 due to usage of alternative stop codons. Our sequencing of the subgroup A2 was from amino acid 28 to end. We observed changes in these sequences leading to gains or loss of N-glycosylation sites. A total of five N-glycosylation sites at positions 30, 52, 145, 152 and 180 were identified on the sequenced G protein. One-hundred-three of the 228 codon positions were polymorphic and up to 5 variants were identified based on sharing a combination of ≥5 signature amino acid residues. There were six sites where amino acid changes led to gain and another eight different sites where changes led to loss of N-glycosylation (Additional file 4: Figure S3). The frequency of gain and loss of N-glycosylation overall was 36 and 56, respectively. Position 180 had one of the most frequent losses in N-glycosylation that occurred in 39 sequences. Overall, from the amino acid changes observed, the pattern of changes clearly demarcated the sequence set into five clusters (Additional file 4: Figure S3).

The HMPV F protein is on average 539 amino acids long. Our F sequencing encompassed 105 codon positions, representing 19.5 % of the entire F protein sequence. Of those that we sequenced, 15 % (17/115) showed amino acid changes, confirming its high degree of conservation. There was no N-glycosylation site observed in the sequenced region of the F protein (Additional file 5: Figure S4).

## Discussion

The epidemiological and evolutionary patterns of circulating strains of HMPV remains poorly documented in most of sub-Saharan Africa. Using an existing framework for childhood pneumonia surveillance at a referral hospital in coastal Kenya we set out to describe HMPV epidemiology as well as its genetic diversity in this region and compared findings to global contemporaneous strains deposited into GenBank.

We found that 4.8 % of childhood pneumonia hospital admissions for the period 2007 to 2011 (inclusive) in Kilifi County Hospital were HMPV positive. Our results fall in the range 3.8 to 15 % [13–15, 20, 23, 48] reported in pediatric hospital admissions in other parts of the world. A previous study in Kenya (albeit in a refugee population) identified HMPV prevalence of 5.7 % [17].

The HMPV infections in KCH admissions were most common in children <6 months (44 %), and 74 % of all HMPV cases occurred in children under 12 months of age, with 84 % of cases presenting with symptoms of severe pneumonia. Substantial disease burden associated with HMPV in the first year of life has been previously reported [7, 13, 16, 22, 49], highlighting the most affected age group and providing a guide on the populations to prioritize in future HMPV vaccine administration.

A seasonal pattern to HPMV positive samples was identified from October of 1 year to April of the next, corresponding to higher temperatures and lower rainfall. This is similar to the seasonal pattern of RSV at the same site [49, 50]. In Dadaab, a refugee camp 500Km north of Kilifi, peak HMPV prevalence occurs in December [17], similar to Kilifi. In other parts of the world, seasonality in HMPV prevalence has been previously reported [14, 15, 17] with peak prevalence either coinciding with the winter season, concurrent or after the RSV epidemic season [13, 15, 20]; alternating between winter and spring [51] or peaking in the late spring-summer months [52] in the northern hemisphere whilst studies in Australia show peak seasons in spring [16] which is concurrent with the RSV peak season. In 2010, no HMPV was detected between April and September. Studies in Europe have similarly shown HMPV prevalence varies from year to year [51, 52].

Three HMPV subgroups A2, B1, B2 were found in Kilifi during the study period; A1 was absent but A2 and B2 occurred over the whole surveillance period whilst B1 was only recorded in the 2007 and 2008 in low numbers. All the samples sequenced for G gene were A2, reflecting the fact that A2 was the predominant subtype in Kilifi in every season/year of the study. Examination of global HMPV sequences in GenBank for the period covered in our study showed low representation of A1, possibly explaining its absence from Kilifi. Interestingly, studies have shown A1 to be dominant in the USA [13] and South Africa [25] and B1 in an Australian study of inpatient admissions of all ages over a 4 year period [16]. Genotype B1 was only detected in 2007 and 2008, and undetectable in the remaining 3 years. Our analysis of comparison sequence data in public databases of the same period showed that B1 was indeed circulating elsewhere in the 3 years that we did not detect it in Kilifi. A 20-year study in the USA reported sporadic detection of B1 genotype [53] and may in part explain the intermittent pattern of occurrence that we observe in Kilifi. Studies covering a longer time period may better resolve the pattern of genotype occurrence. The identification of three subgroups of HMPV was possible using only F sequence data and not the G sequence data possibly owing to the larger number of samples successfully sequenced for the F.

Co-circulation of multiple lineages of HMPV has been previously reported [7, 14, 53, 54]. Furthermore, dominant strains may vary in different seasons and locations [7, 36, 55] and genotypes may dominate in 1 year then be replaced by another the subsequent year [16, 30, 53]. In Kilifi, our data showed a contrasting scenario with one subgroup A2 dominant in all seasons, with the other subgroups especially B2 co-circulating in lower numbers. Long-term surveillance to ascertain if there is genotype replacement in the subsequent years after the study will be important in determining genotype patterns in Kilifi. The dissimilarity between the distribution of genotypes in circulation in Kilifi relative to the global pattern, comparing year by year, supports the hypothesis that HMPV migrates across the world at a relatively slower rate compared to other respiratory viruses like Influenza A to allow the existence of localized genotype replacement patterns.

The G gene is the most variable gene in the HMPV genome [10, 56]. It has been suggested that frequent variation in the G gene may be a strategy to evade the host immune system selective pressure [57]. We found that the G protein evolutionary rate was three times higher in the region we analyzed compared to the F protein evolutionary rate. Overall the diversity observed in the G protein sequences was far higher as compared to the F protein sequences. Although the all G sequences we obtained were of A2 subgroup, based on the phylogenetic clustering, bootstrap support and amino acid change patterns, we could classify our A2 subgroup viruses into four further clusters.

Amplification and sequencing of the G protein did not succeed for more than half of our HMPV positive samples. About 60 samples failed at the G PCR amplification stage and a further 30 failed at the nucleotide sequencing stage. In the end we only obtained sequences for subgroup A2. It is possible that this was caused by insufficient match of our primers to the circulating variants/subgroups thus impeding amplification and sequencing. This low recovery rate of the HMPV G protein sequences limited our study power to fully understand genotypes and variants that circulated in Kilifi over the study period. An alternative explanation for the PCR/sequencing failures is possible RNA degradation as the study used archived material.

The HMPV F gene was determined to be less diverse which concurs with previous findings and pneumovirus F protein diversity in general [8]. Furthermore, 52 % (64/123) of the F gene sequences were determined as 100 % identical to a sequences in the remaining set whilst only 5.3 % (3/56) of the G gene sequences were identical to each other. This suggests that to tease out any differences between these sequences that are 100 % F gene identical, sequencing another gene for instance the G gene or even the whole genome will be necessary.

Whilst there were no N-glycosylation sites detected in the F protein sequenced and only few amino acid changes observed, the G protein had more sites where there was loss of N-glycosylation and also had several amino acid changes owing to the more nucleotide sequence diversity observed. The F portion we sequenced does not encompass the three potential N-glycosylation sites at positions 57, 127 and 353 that have been previously described for HMPV [58].

Many of the characteristic epidemiological and evolutionary patterns observed in this study for HMPV mirror the findings previously reported for RSV from the Kilifi population. For both viruses, the highest disease burden is in the paediatric population occurring during early infancy (though HMPV burden is overall smaller) [49]. Both RSV and HMPV show an annual seasonal pattern with peak activity months well overlapped [49, 50] and multiple genotypes occur during epidemics [50, 59]. Further we show that like for RSV, analysis of the G protein encoding region distinguishes better the variability of strains occurring across epidemics than the F [60]. Nonetheless, a few differences can still be picked in their patterns. Firstly, group/subgroup temporal dominance or replacement is clearer with RSV than HMPV [59]. Secondly, the substitution rate observed in HMPV G appeared much higher than estimated for RSV G [61]. Thirdly, most of the genetic variants in RSV occur over a single epidemic and disappear but for HMPV, variants seem to persist for more than a single epidemic before disappearing [50]. To provide further new insights, future studies should undertake whole genome study of these viruses and analyze specimens collected over a longer time period and across multiple sites in Kenya and Africa for a better understanding of the transmission, evolution and persistence mechanisms of these important human pathogens.

There are a number of limitations of this study that should be considered when interpreting the results. Fewer G gene sequences were obtained from the study, relative to F, and this could be the reason that only the A2 variant from the worldwide pool was identified. There was a change through time in the selection of samples for testing based on residency status, which would have resulted in a wider catchment area for 2010-11 than earlier. However, the low level of temporal clustering observed suggests the samples to be drawn from a similar pool of variants. As noted in previous reports from this surveillance [27, 43, 49], collection of nasal specimens from children with life-threatening features is a continual challenge that could bias estimates of prevalence and variant composition.

## Conclusions

In conclusion, we report on 5 years of epidemiological surveillance and on circulating HMPV genotypes in the coastal Kenyan location of Kilifi. The study reveals three of the four globally circulating HMPV subgroups, with the same dominance of A2 subgroup, but with annual variation in subgroup prevalence not mirrored in the wider global dataset, and little temporal clustering of the subgroups A2 in this region of the world. The dissimilarity between the distribution of genotypes in circulation in Kilifi relative to the global pattern, may suggest that slow global migration of HMPV allows the existence of localized genotype replacement patterns. One major peak season of HMPV was observed, and prevalence was universally highest in infants, especially those <6 months of age. Furthermore, HMPV cases occurred in roughly annual outbreaks, with a prevalence of around 5 % in severe and very severe pneumonia paediatric admissions to the County hospital.

## Abbreviations

HMPV, human metapneumovirus; RT-PCR, real time polymerase chain reaction; KCH, Kilifi County Hospital; HPD, highest posterior density.

## Additional files

Additional file 1: Table S1. Reference HMPV sequences for F gene from GenBank used for phylogenetic analyses. **Table S2.** Reference HMPV sequences for G gene from GenBank used for phylogenetic analyses. **Table S3.** GenBank accession numbers for F gene HMPV sequences from Kilifi, Kenya 2007-11 generated in this study. **Table S4.** GenBank accession numbers for G gene HMPV sequences from Kilifi, Kenya 2007-11, generated in this study. (DOCX 38 kb) Additional file 2: Figure S1. ML phylogeny of F and G gene sequences colored by epidemic, from Kilifi 2007-11. Panel A. Using 123 F gene sequences. Panel B. Using 56 G gene sequences. All viruses in Panel B are subgroup A2. (PPTX 175 kb) Additional file 3: Figure S2. A. Temporal distribution of HMPV subgroups by month isolated from Kilifi County Hospital in the 5 years of the study (2007-11). Numbers at the bottom indicate number of genotypes each year determined by a combined F and G gene sequencing (total of 123 out of the 160 positives) in Kilifi, Kenya in the period 2007–2011. (PPTX 86 kb) Additional file 4: Figure S3. An amino acid sequence alignment predicted from the 53 unique G gene nucleotide sequences from Kilifi, Coastal Kenya, 2007-11. N-glycosylation sites have been highlighted: pink colour for the identified sites; blue colour for loss and yellow for gains of N-glycosylation sites. (PPTX 445 kb) Additional file 5: Figure S4. An amino acid sequence alignment predicted from 49 unique F gene nucleotide sequences from Kilifi, Coastal Kenya, 2007-11. (PPTX 259 kb)

## References

[1] Van den Hoogen BG, de Jong JC, Groen J, Kuiken T, de Groot R, Fouchier RA, Osterhaus AD. A newly discovered human metapneumovirus isolated from young children with respiratory tract disease. Nat Med. 2001;7(6):719–24. 10.1038/89098 PMC7095854 11385510

[2] Williams JV (2005). Human metapneumovirus: an important cause of respiratory disease in children and adults. Curr Infect Dis Rep.

[3] Wang Y, Ji W, Chen Z, Yan YD, Shao X, Xu J. Comparison of severe pneumonia caused by Human metapneumovirus and respiratory syncytial virus in hospitalized children. Indian J Pathol Microbiol. 2014;57(3) doi: 10.4103/0377-4929.138735.):413%E2%80%93417 10.4103/0377-4929.138735 25118733

[4] Akhras N, Weinberg JB, Newton D (2010). Human metapneumovirus and respiratory syncytial virus: subtle differences but comparable severity. Infectious Disease Reports.

[5] van den Hoogen BG, Bestebroer TM, Osterhaus AD, Fouchier RA (2002). Analysis of the genomic sequence of a human metapneumovirus. Virology.

[6] Skiadopoulos MH, Biacchesi S, Buchholz UJ, Amaro-Carambot E, Surman SR, Collins PL, Murphy BR. Individual contributions of the human metapneumovirus F, G, and SH surface glycoproteins to the induction of neutralizing antibodies and protective immunity. Virology. 2006;345(2):492–501. 10.1016/j.virol.2005.10.016 16300813

[7] Banerjee S, Sullender WM, Choudekar A, John C, Tyagi V, Fowler K, Lefkowitz EJ. Detection and genetic diversity of human metapneumovirus in hospitalized children with acute respiratory infections in India. J Clin Virol. 2011;83:1799–810. 10.1002/jmv.22176 PMC4412166 21837798

[8] van den Hoogen BG, Herfst S, Sprong L, Cane PA, Forleo-Neto E, de Swart RL, Osterhaus ADME, Fouchier RAM. Antigenic and genetic variability of human metapneumoviruses. Emerg Infect Dis. 2004;10(4):658–66. 10.3201/eid1004.030393 PMC3323073 15200856

[9] Bastien N, Liu L, Ward D, Taylor T, Li Y (2004). Genetic variability of the G glycoprotein gene of human metapneumovirus. J Clin Microbiol.

[10] Biacchesi S, Skiadopoulos MH, Boivin G, Hanson CT, Murphy BR, Collins PL (2003). Genetic diversity between human metapneumovirus subgroups. Virology.

[11] Ishiguro N, Ebihara T, Endo R, Ma X, Kikuta H, Ishiko H, Kobayashi K. High genetic diversity of the attachment (G) protein of human metapneumovirus. J Clin Microbiol. 2004;42(8):3406–14. 10.1128/JCM.42.8.3406-3414.2004 PMC497604 15297475

[12] Schildgen V, van den Hoogen B, Fouchier R, Tripp RA, Alvarez R, Manoha C, Williams J, Schildgen O. Human metapneumovirus: lessons learned over the first decade. Clin Microbiol Rev. 2011;24(4):734–54. 10.1128/CMR.00015-11 PMC3194831 21976607

[13] Williams JV, Edwards KM, Weinberg GA, Griffin MR, Hall CB, Zhu Y, Szilagyi PG, Wang CK, Yang C-F, Silva D, et al. Population-based incidence of human metapneumovirus infection among hospitalized children. J Infect Dis. 2010;201:1890–8. 10.1086/652782 PMC2873123 20446850

[14] Bastien N, Ward D, Van Caeseele P, Brandt K, Lee SHS, McNabb G, Klisko B, Chan E, Li Y. Human metapneumovirus infection in the Canadian population. J Clin Microbiol. 2003;41(10):4642–6. 10.1128/JCM.41.10.4642-4646.2003 PMC254302 14532196

[15] Mullins JA, Erdman DD, Weinberg GA, Edwards K, Hall CB, Walker FJ, Iwane M, Anderson LJ. Human metapneumovirus infection among children hospitalized with acute respiratory illness. Emerg Infect Dis. 2004;10(4). 10.3201/eid1004.030555 PMC3323105 15200863

[16] Mackay IM, Bialasiewicz S, Jacob KC, McQueen E, Arden KE, Nissen MD, Sloots TP. “Genetic diversity of human metapneumovirus over 4 consecutive years in Australia”. J Infect Dis. 2006;193(12):1630–3. 10.1086/504260 16703505

[17] Ahmed JA, Katz MA, Auko E, Kariuki Njenga M, Weinberg M, Kapella BK, Burke H, Nyoka R, Gichangi A, Waiboci LW, et al. Epidemiology of respiratory viral infections in two long-term refugee camps in Kenya, 2007–2010. BMC Infect Dis. 2012;12(7). 10.1186/1471-2334-12-7 PMC3398263 22251705

[18] Feikin DR, Kariuki NM, Bigogo G, Aura B, Aol G, Audi A, Jagero G, Muluare PO, Gikunju S, Nderitu L, et al. Etiology and Incidence of Viral and Bacterial Acute Respiratory Illness among Older Children and Adults in Rural Western Kenya, 2007–2010. PLoS One. 2012;7(8). 10.1371/journal.pone.0043656 PMC3427162 22937071

[19] Arnott A, Vong S, Sek M, Naughtin M, Beauté J, Rith S, Guillard B, Deubel V, Buchy P. Genetic variability of human metapneumovirus amongst an all ages population in Cambodia between 2007 and 2009. Infect Genet Evol. 2011. doi:10.1016/j.meegid.2011.01.016. 10.1016/j.meegid.2011.01.016 PMC7106057 21292032

[20] Boivin G, De Serres G, Côté S, Gilca R, Abed Y, Rochette L, Bergeron MG, Déry P. Human metapneumovirus infections in hospitalized children. Emerg Infect Dis. 2003;9(6):634–40. 10.3201/eid0906.030017 PMC3000156 12781001

[21] Cuevas LE, Nasser AMB, Dove W, Gurgel RQ, Greensill J, Hart CA (2003). Human metapneumovirus and respiratory syncytial virus, Brazil. Emerg Infect Dis.

[22] Edwards KM, Yuwei X, Griffin MR, Weinberg G, Hall CB, Szilagyi PG, Staat MD, Iwane M, Prill MM, Williams JV. Burden of human metapneumovirus infection in young children. N Engl J Med. 2013;368(7):633–43. 10.1056/NEJMoa1204630 PMC3662802 23406028

[23] Jartti T, van den Hoogen B, Garofalo RP, Osterhaus AD, Ruuskanen O (2002). Metapneumovirus and acute wheezing in children. Lancet.

[24] Loo LH, Tan BH, Ng LM, Tee NWS, Lin RTP, Sugrue RJ (2007). Human metapneumovirus in children, Singapore. Emerg Infect Dis.

[25] Ludewick HP, Abed Y, van Nierkek N, Boivin G, Klugman KP, Madhi SA (2005). Human metanpneumovirus genetic variability, South Africa. Emerg Infect Dis.

[26] Zhang C, Du L-N, Zhang Z-Y, Qin X, Yang X, Liu P, Chen X, Zhao Y, Liu EM, Zhao X-D. Detection and genetic diversity of human metapneumovirus in hospitalized children with acute respiratory infections in southwest China. J Clin Microbiol. 2012;50(8):2714–9. 10.1128/JCM.00809-12 PMC3421497 22692746

[27] Berkley JA, Munywoki P, Ngama M, Kazungu S, Abwao J, Bett A, Lassauniére R, Kresfelder T, Cane PA, Venter M, et al. Viral etiology of severe pneumonia among Kenyan young infants and children. JAMA. 2010;303(20):2051–7. 10.1001/jama.2010.675 PMC2968755 20501927

[28] Hammit L, Kazungu S, Welch S, Bett A, Onyango C, Gunson R, Scott J, Nokes D. Added value of an oropharyngeal sab in detection of viruses in children hospitalized with lower respiratory tract infection. J Clin Microbiol. 2011;49:2318–20. 10.1128/JCM.02605-10 PMC3122752 21490188

[29] Hammitt LL, Kazungu S, Morpeth SC, Gibson DG, Mvera B, Brent AJ, Mwarumba S, Onyango CO, Bett A, Akech DO, et al. A preliminary study of pneumonia etiology among hospitalized children in Kenya. Clin Infect Dis. 2012;54(S2):S190–9. 10.1093/cid/cir1071 PMC3297554 22403235

[30] Kim HR, Cho AR, Lee M-K, Yun SW, Kim T-H (2012). Genotype variability and clinical features of human metapneumovirus isolated from Korean children, 2007 to 2010. Journal of Medical Diagnostics.

[31] Gaunt ER, Jansen RR, Poovorawan Y, Templeton KE, Toms GL, Simmonds P (2011). Molecular epidemiology and evolution of human respiratory syncytial virus and human metapneumovirus. PLoS One.

[32] Xiao NG, Zhang B, Xie ZP, Zhou QH, Zhang RF, Zhong LL, Ding XF, Li J, Song JR, Gao HC, et al. Prevalence of human metapneumovirus in children with acute lower respiratory infection in Changsha, China. J Med Virol. 2013;85(3). 10.1002/jmv.23501 PMC7166472 23296388

[33] Vicente D, Montes M, Cilla G, Perez-Yarza EG, Perez-Trallero E (2006). Differences in clinical severity between genotype A and genotype B human metapneumovirus infection in children. Clin Infect Dis.

[34] Huck B, Scharf G, Neumann-Heifelin D, Puppe W, Weigl J, Falcone V (2006). Novel human metapneumovirus sublineage. Emerg Infect Dis.

[35] Li J, Ren L, Guo L, Xiang Z, Paranhos-Baccala G, Vernet G, Wang J. Evolutionary dynamics analysis of human metapneumovirus subtype A2: genetic evidence for its dominant epidemic. PLoS One 2012;7(3). 10.1371/journal.pone.0034544 PMC3316673 22479641

[36] Peret TC, Boivin G, Li Y, Couillard M, Humphrey C, Osterhaus AD, Erdman DD, Anderson LJ. Characterization of human metapneumo- viruses isolated from patients in North America. J Infect Dis. 2002;185:1660–3. 10.1086/340518 PMC7109943 12023774

[37] Principi N, Esposito S (2014). Paediatric human metapneumovirus infection: epidemiology, prevention and therapy. J Clin Virol.

[38] Cox RG, Erickson JJ, Hastings AK, Becker JC, Johnson M, Craven RE, Tollefson SJ, Boyd KL, Williams JV. Human metapneumovirus virus-like particles induce protective B and T cell responses in a mouse model. J Virol. 2014;88(11):6368–79. 10.1128/JVI.00332-14 PMC4093842 24672031

[39] Herfst S, Schrauwen EJ, de Graaf M, van Amerongen G, van den Hoogen BG, de Swart RL, Osterhaus AD, Fouchier RA. Immunogenicity and efficacy of two candidate human metapneumovirus vaccines in cynomolgus macaques. Vaccine. 2008;5(26(33)):4224–30. 10.1016/j.vaccine.2008.05.052 18585830

[40] Lévy C, Aerts L, Hamelin MÈ, Granier C, Szécsi J, Lavillette D, Boivin G, Cosset FL. Virus-like particle vaccine induces cross-protection against human metapneumovirus infections in mice. Vaccine. 2013;7(31(25)):2778–85. 10.1016/j.vaccine.2013.03.051 23583815

[41] WHO (1990). Programme for the control of acute respiratory infections. Acute respiratory infections in children: case management in small hospitals in developing countries. A manual for doctors and other senior health workers.

[42] Scott JA, Bauni E, Moisi JC, Ojal J, Gatakaa H, Nyundo C, Molyneux CS, Kombe F, Tsofa B, Marsh K, et al. Profile: The Kilifi Health and Demographic Surveillance System (KHDSS). Int J Epidemiol. 2012;41(3):650–7. 10.1093/ije/dys062 PMC3396317 22544844

[43] Onyango OC, Njeru R, Kazungu S, Achilla R, Bulimp W, Welch SR (2012). Influenza surveillance among children with pneumonia admitted to a district hospital in coastal Kenya, 2007–2010. J Infect Dis.

[44] Nokes DJ, Abwao J, Pamba A, Peenze I, Dewar J, Maghenda JK, Gatakaa H, Bauni E, Scott JA, Maitland K, et al. Incidence and clinical characteristics of group A rotavirus infections among children admitted to hospital in Kilifi, Kenya. PLoS Med. 2008;5(e153). 10.1371/journal.pmed.0050153 PMC2488191 18651787

[45] Banerjee S, Bharaj P, Sullender W, Kabra SK, Broor S (2007). Human metapneumovirus infection among children with acure respiratoty infections seen in a large referral hospital in India. J Clin Virol.

[46] Katoh S (2013). MAFFT multiple sequence alignment software version 7: improvements in performance and usability. Mol Biol Evol.

[47] Tamura K, Peterson D, Peterson N, Stecher G, Nei M, Kumar S (2011). MEGA5: molecular evolutionary genetics analysis using maximum likelihood, evolutionary distance, and maximum parsimony methods. Mol Biol Evol.

[48] Falsey AR, Erdman D, Anderson LJ, Walsh EE (2003). Human metapneumovirus infections in young and elderly adults. J Infect Dis.

[49] Nokes DJ, Ngama MJ, Bett A, Abwao J, Munywoki P, English M, Scott JAG, Cane PA, Medley GF. Incidence and severity of respiratory syncytial virus pneumonia in rural Kenyan children identified through hospital surveillance. Clin Infect Dis. 2009;49(9):1341–9. 10.1086/606055 PMC2762474 19788358

[50] Agoti CN, Otieno JR, Munywoki PK, Mwihuri AG, Cane PA, Nokes DJ, Kellam P, Cotten M. Local evolutionary patterns of human respiratory syncytial virus derived from whole-genome sequencing. J Virol. 2015;89:3444–54. 10.1128/JVI.03391-14 PMC4403408 25609811

[51] Aberle SW, Aberle JH, Sandhofer MJ, Pracher E, Popow-Kraupp T (2008). Biennial spring activity of human metapneumovirus in Austria. Pediatr Infect Dis J.

[52] Aberle JH, Aberle SW, Redlberger-Fritz M, Sandhofer MJ, Popow-Kraupp T (2010). Human metapneumovirus subgroup changes and seasonality during epidemics. Pediatr Infect Dis J.

[53] Williams JV, Wang CK, Yang C-F, Tollefson SJ, House FS, Heck JM, Chu M, Brown JB, Lintao LD, Quinto JD, et al. The role of human metapneumovirus in upper respiratory tract infections in children: a 20-year experience. J Infect Dis. 2006;193:387–95. 10.1086/499274 PMC1586246 16388486

[54] Papenburg J, Carbonneau J, Isabel S, Bergeron MG, Williams JV, De Serres G, Hamelin MÈ, Boivin G. Genetic diversity and molecular evolution of the major human metapneumovirus surface glycoproteins over a decade. J Clin Virol. 2013;58(3):541–7. 10.1016/j.jcv.2013.08.029 24041471

[55] Peret TC, Hall BC, Schnabel KC, Golub JA, Anderson LJ (1998). Circulation patterns of genetically distinct group A and B strains of human respiratoty syncytial virus in a community. J Gen Virol.

[56] Piyaratna R, Tollefson SJ, Williams JV (2011). Genomic analysis of four human metapneumovirus prototypes. Virus Res.

[57] Kahn JS (2006). Epidemiology of human metapneumovirus. Clin Microbiol Rev.

[58] Schowalter RM, Smith SE, Dutc RE (2006). Characterization of human metapneumovirus F protein-promoted membrane fusion: critical roles for proteolytic processing and low pH. J Virol.

[59] Scott PD, Ochola R, Ngama M, Okiro EA, Nokes DJ, Medley GF, Cane PA. Molecular epidemiology of respiratory syncytial virus in Kilifi district, Kenya. J Med Virol. 2004;74(2):344–54. 10.1002/jmv.20183 15332285

[60] Agoti CN, Mwihuri AG, Sande CJ, Onyango CO, Medley GF, Cane PA, Nokes DJ. Genetic relatedness of infecting and reinfecting respiratory syncytial virus strains identified in a birth cohort from rural Kenya. J Infect Dis. 2012;206(10):1532–41. 10.1093/infdis/jis570 PMC3475639 22966119

[61] Agoti CN, Mayieka LM, Otieno JR, Ahmed JA, Fields BS, Waiboci LW, Nyoka R, Eidex RB, Marano N, Burton W, et al. Examining strain diversity and phylogeography in relation to an unusual epidemic pattern of respiratory syncytial virus (RSV) in a long-term refugee camp in Kenya. BMC Infect Dis. 2014;14(178). 10.1186/1471-2334-14-178 PMC4021307 24690157

