# Supplementary material for: Human metapneumovirus epidemiological and evolutionary patterns in Coastal Kenya, 2007-11
Source: BMC Infect Dis. 2016 Jun 17;16:301. doi: 10.1186/s12879-016-1605-0 (PMC4912817; doi:10.1186/s12879-016-1605-0)
Supplement: Additional file 1: Table S1. — Reference HMPV sequences for F gene from GenBank used for phylogenetic analyses. Table S2. Reference HMPV sequences for G gene from GenBank used for phylogenetic analyses. Table S3. GenBank accession numbers for F gene HMPV sequences from Kilifi, Kenya 2007-11 generated in this study. Table S4. GenBank accession numbers for G gene HMPV sequences from Kilifi, Kenya 2007-11, generated in this study. (DOCX 38 kb) [file 12879_2016_1605_MOESM1_ESM.docx]

Table S1: Reference HMPV sequences for F gene from GenBank used for Phylogenetic analyses

| **Sequence Identity** | **Accession number** | **Date** |
| --- | --- | --- |
| Japan/AB618741/18-Dec-2007 | AB618741 | 18-Dec-07 |
| Japan/AB618743/18-Dec-2007 | AB618743 | 18-Dec-07 |
| Japan/AB618749/18-Feb-2008 | AB618749 | 18-Feb-08 |
| Japan/AB618758/22-Feb-2010 | AB618758 | 22-Feb-10 |
| Japan/AB618760/23-Feb-2010 | AB618760 | 23-Feb-10 |
| Japan/AB693956/17-Mar-2008 | AB693956 | 17-Mar-08 |
| Japan/AB693958/15-Apr-2008 | AB693958 | 15-Apr-08 |
| Netherlands/FJ168779/A2/02-Apr-2000 | FJ168779 | 2-Apr-00 |
| Rwanda/HM197719/01-Jul-2008 | HM197719 | 1-Jul-08 |
| Egypt/JQ041674/19-Jan-2008 | JQ041674 | 19-Jan-08 |
| Egypt/JQ041675/26-Jan-2008 | JQ041675 | 26-Jan-08 |
| Egypt/JQ041679/28-Jan-2008 | JQ041679 | 28-Jan-08 |
| Egypt/JQ041680/28-Jan-2008 | JQ041680 | 28-Jan-08 |
| Egypt/JQ041681/28-Jan-2008 | JQ041681 | 28-Jan-08 |
| Thailand/JQ181560/01-Sep-2011 | JQ181560 | 1-Sep-11 |
| Thailand/JQ181562/01-Sep-2011 | JQ181562 | 1-Sep-11 |
| Thailand/JQ181563/01-Sep-2011 | JQ181563 | 1-Sep-11 |
| Thailand/JQ181565/01-Sep-2011 | JQ181565 | 1-Sep-11 |
| Thailand/JQ181572/01-Sep-2011 | JQ181572 | 1-Sep-11 |
| Thailand/JQ181573/01-Sep-2011 | JQ181573 | 1-Sep-11 |
| Thailand/JQ181584/01-Sep-2011 | JQ181584 | 1-Sep-11 |
| Thailand/JQ745050/01-Sep-2011 | JQ745050 | 1-Sep-11 |
| Thailand/JQ745051/01-Sep-2011 | JQ745051 | 1-Sep-11 |
| Thailand/JQ745052/01-Sep-2011 | JQ745052 | 1-Sep-11 |
| Thailand/JQ745055/01-Sep-2011 | JQ745055 | 1-Sep-11 |
| Thailand/JQ745056/01-Sep-2011 | JQ745056 | 1-Sep-11 |
| Thailand/JQ745060/01-Sep-2011 | JQ745060 | 1-Sep-11 |
| Thailand/JQ745061/01-Sep-2011 | JQ745061 | 1-Sep-11 |
| Thailand/JQ745063/01-Sep-2011 | JQ745063 | 1-Sep-11 |
| Thailand/JQ745064/01-Sep-2011 | JQ745064 | 1-Sep-11 |
| Thailand/JQ745066/01-Sep-2011 | JQ745066 | 1-Sep-11 |
| Thailand/JQ745067/01-Sep-2011 | JQ745067 | 1-Sep-11 |
| Thailand/JQ745068/01-Sep-2011 | JQ745068 | 1-Sep-11 |
| Thailand/JQ745069/01-Sep-2011 | JQ745069 | 1-Sep-11 |
| Thailand/JQ745071/01-Sep-2011 | JQ745071 | 1-Sep-11 |
| Thailand/JQ745074/01-Sep-2011 | JQ745074 | 1-Sep-11 |
| Thailand/JQ745075/01-Sep-2011 | JQ745075 | 1-Sep-11 |
| Thailand/JQ745078/01-Sep-2011 | JQ745078 | 1-Sep-11 |
| Thailand/JQ745079/01-Sep-2011 | JQ745079 | 1-Sep-11 |
| Thailand/JQ745080/01-Sep-2011 | JQ745080 | 1-Sep-11 |
| Thailand/JQ745081/01-Sep-2011 | JQ745081 | 1-Sep-11 |
| Thailand/JQ745085/01-Sep-2011 | JQ745085 | 1-Sep-11 |
| Thailand/JQ745089/01-Sep-2011 | JQ745089 | 1-Sep-11 |
| Thailand/JQ745090/01-Sep-2011 | JQ745090 | 1-Sep-11 |
| Thailand/JQ745093/01-Sep-2011 | JQ745093 | 1-Sep-11 |
| Thailand/JQ745094/01-Sep-2011 | JQ745094 | 1-Sep-11 |
| Nairobi/JQ888113/03-Apr-2008 | JQ888113 | 3-Apr-08 |
| Nairobi/JQ888114/10-Apr-2008 | JQ888114 | 10-Apr-08 |
| Nairobi/JQ888115/10-Apr-2008 | JQ888115 | 10-Apr-08 |
| Nairobi/JQ888116/17-Apr-2008 | JQ888116 | 17-Apr-08 |
| Nairobi/JQ888118/16-Jun-2008 | JQ888118 | 16-Jun-08 |
| India/KC731505/15-Aug-2009 | KC731505 | 15-Aug-09 |
| India/KC731508/30-Dec-2010 | KC731508 | 30-Dec-10 |
| India/KC731509/11-Aug-2011 | KC731509 | 11-Aug-11 |
| India/KC731510/20-Aug-2011 | KC731510 | 20-Aug-11 |
| India/KC731514/02-Sep-2011 | KC731514 | 2-Sep-11 |
| India/KC731516/06-Sep-2011 | KC731516 | 6-Sep-11 |
| India/KC731518/29-Sep-2011 | KC731518 | 29-Sep-11 |
| India/KC731519/21-Jan-2011 | KC731519 | 21-Jan-11 |
| India/KC731525/15-Mar-2011 | KC731525 | 15-Mar-11 |
| India/KC731527/18-Jan-2011 | KC731527 | 18-Jan-11 |
| Canada/KF192723/23-Mar-2007 | KF192723 | 23-Mar-07 |
| Canada/KF192724/10-Feb-2007 | KF192724 | 10-Feb-07 |
| Canada/KF192725/24-Feb-2007 | KF192725 | 24-Feb-07 |
| Canada/KF192727/22-Apr-2007 | KF192727 | 22-Apr-07 |
| Canada/KF192729/07-May-2007 | KF192729 | 7-May-07 |
| Canada/KF192730/29-Feb-2008 | KF192730 | 29-Feb-08 |
| Canada/KF192731/08-Mar-2008 | KF192731 | 8-Mar-08 |
| Canada/KF192733/24-Mar-2008 | KF192733 | 24-Mar-08 |
| Canada/KF192735/14-Jan-2009 | KF192735 | 14-Jan-09 |
| Canada/KF192738/02-Apr-2009 | KF192738 | 2-Apr-09 |
| Canada/KF192740/09-Apr-2009 | KF192740 | 9-Apr-09 |
| Canada/KF192742/17-Jan-2009 | KF192742 | 17-Jan-09 |
| Canada/KF192744/18-Apr-2009 | KF192744 | 18-Apr-09 |
| Canada/KF192747/05-Feb-2010 | KF192747 | 5-Feb-10 |
| Canada/KF192748/09-Feb-2010 | KF192748 | 9-Feb-10 |
| Canada/KF192750/09-Feb-2010 | KF192750 | 9-Feb-10 |
| Canada/KF192751/16-Feb-2010 | KF192751 | 16-Feb-10 |
| Canada/KF192752/05-Mar-2010 | KF192752 | 5-Mar-10 |
| Canada/KF192754/23-Mar-2010 | KF192754 | 23-Mar-10 |
| Canada/KF192755/31-Jan-2010 | KF192755 | 31-Jan-10 |
| Canada/KF192756/08-Feb-2010 | KF192756 | 8-Feb-10 |
| Canada/KF192757/03-Mar-2010 | KF192757 | 3-Mar-10 |
| Canada/KF192758/03-Mar-2010 | KF192758 | 3-Mar-10 |
| Canada/KF192759/09-Mar-2010 | KF192759 | 9-Mar-10 |
| Canada/KF192760/08-Mar-2010 | KF192760 | 8-Mar-10 |
| Canada/KF192763/29-Mar-2010 | KF192763 | 29-Mar-10 |
| Canada/KF192769/09-Mar-2007 | KF192769 | 9-Mar-07 |
| Canada/KF192770/01-Feb-2007 | KF192770 | 1-Feb-07 |
| Canada/KF192773/11-Dec-2007 | KF192773 | 11-Dec-07 |
| Canada/KF192774/10-Jan-2008 | KF192774 | 10-Jan-08 |
| Canada/KF192775/05-Feb-2008 | KF192775 | 5-Feb-08 |
| Canada/KF192780/27-Feb-2008 | KF192780 | 27-Feb-08 |
| Canada/KF192781/27-Feb-2008 | KF192781 | 27-Feb-08 |
| Canada/KF192784/15-Apr-2008 | KF192784 | 15-Apr-08 |
| Canada/KF192788/02-Apr-2008 | KF192788 | 2-Apr-08 |
| Canada/KF192793/01-Apr-2009 | KF192793 | 1-Apr-09 |
| Canada/KF192794/27-Feb-2009 | KF192794 | 27-Feb-09 |
| Canada/KF192796/11-Mar-2009 | KF192796 | 11-Mar-09 |
| Canada/KF192797/15-Apr-2009 | KF192797 | 15-Apr-09 |
| Canada/KF192798/15-Jan-2010 | KF192798 | 15-Jan-10 |
| Canada/KF192799/12-Mar-2010 | KF192799 | 12-Mar-10 |
| Canada/KF192800/01-Feb-2010 | KF192800 | 1-Feb-10 |
| Canada/KF192801/04-Feb-2010 | KF192801 | 4-Feb-10 |
| Peru/KJ627377/24-May-2010 | KJ627377 | 24-May-10 |
| Peru/KJ627380/08-Jun-2010 | KJ627380 | 8-Jun-10 |
| Peru/KJ627381/15-Jun-2010 | KJ627381 | 15-Jun-10 |
| Peru/KJ627382/18-Jan-2011 | KJ627382 | 18-Jan-11 |
| Peru/KJ627384/08-Feb-2010 | KJ627384 | 8-Feb-10 |
| Peru/KJ627385/21-Nov-2009 | KJ627385 | 21-Nov-09 |
| Peru/KJ627386/03-Sep-2008 | KJ627386 | 3-Sep-08 |
| Peru/KJ627387/14-Jan-2010 | KJ627387 | 14-Jan-10 |
| Peru/KJ627391/07-Oct-2008 | KJ627391 | 7-Oct-08 |
| Peru/KJ627398/06-May-2011 | KJ627398 | 6-May-11 |
| Peru/KJ627401/10-Feb-2011 | KJ627401 | 10-Feb-11 |
| Peru/KJ627402/06-May-2009 | KJ627402 | 6-May-09 |
| Peru/KJ627406/24-Feb-2010 | KJ627406 | 24-Feb-10 |
| Peru/KJ627425/18-Apr-2011 | KJ627425 | 18-Apr-11 |
| Peru/KJ627431/20-Apr-2009 | KJ627431 | 20-Apr-09 |
| Peru/KJ627432/30-Nov-2009 | KJ627432 | 30-Nov-09 |
| Peru/KJ627435/22-May-2009 | KJ627435 | 22-May-09 |
| Peru/KJ627437/21-Jul-2011 | KJ627437 | 21-Jul-11 |
| Germany/HQ456635/A2b/23-Feb-2010 | HQ456635 | 23-Feb-10 |
| Peru/KJ627424/A2b/28-Jan-2009 | KJ627424 | 28-Jan-09 |
| HQ456571/Germany/A2a/21-Dec-2003 | HQ456571 | 21-Dec-03 |
| HQ456557/Germany/A1/31-Jan-2002 | HQ456557 | 31-Jan-02 |
| HQ456625/Germany/B2/21-Dec-2009 | HQ456625 | 21-Dec-09 |
| Australia/KF530173/B1/06-Jul-2004 | KF530173 | 6-Jul-04 |

Table S2: Reference HMPV sequences for G gene from GenBank used for Phylogenetic analyses

| **Sequence Identity** | **Accession number** | **Date** |
| --- | --- | --- |
| Canada/KF178997/23-Mar-2010 | KF178997 | 23-Mar-10 |
| Canada/KF178985/03-Mar-2010 | KF178985 | 3-Mar-10 |
| Canada/KF178988/14-Mar-2010 | KF178988 | 14-Mar-10 |
| India/KC731502/10-Aug-2009 | KC731502 | 10-Aug-09 |
| India/HQ599215/01-Jul-2009 | HQ599215 | 1-Jul-09 |
| India/HQ599217/01-Jul-2009 | HQ599217 | 1-Jul-09 |
| Canada/KF178991/02-Feb-2010 | KF178991 | 2-Feb-10 |
| India/KC731491/19-Sep-2009 | KC731491 | 19-Sep-09 |
| India/KC731490/18-Sep-2009 | KC731490 | 18-Sep-09 |
| India/HQ599213/01-Jul-2007 | HQ599213 | 1-Jul-07 |
| India/KC731494/26-Sep-2009 | KC731494 | 26-Sep-09 |
| Canada/KF178964/10-Feb-2007 | KF178964 | 10-Feb-07 |
| Canada/KF178972/17-Mar-2008 | KF178972 | 17-Mar-08 |
| Canada/KF178967/22-Apr-2007 | KF178967 | 22-Apr-07 |
| Peru/KJ627403/28-Dec-2009 | KJ627403 | 28-Dec-09 |
| Peru/KJ627409/26-Nov-2009 | KJ627409 | 26-Nov-09 |
| Peru/KJ627386/03-Sep-2008 | KJ627386 | 3-Sep-08 |
| Peru/KJ627399/11-Oct-2010 | KJ627399 | 11-Oct-10 |
| Peru/KJ627387/14-Jan-2010 | KJ627387 | 14-Jan-10 |
| Peru/KJ627380/08-Jun-2010 | KJ627380 | 8-Jun-10 |
| Peru/KJ627377/24-May-2010 | KJ627377 | 24-May-10 |
| Peru/KJ627411/25-May-2010 | KJ627411 | 25-May-10 |
| India/HQ599202/01-Jul-2007 | HQ599202 | 1-Jul-07 |
| Canada/KF178994/05-Mar-2010 | KF178994 | 5-Mar-10 |
| Peru/KJ627422/07-Nov-2009 | KJ627422 | 7-Nov-09 |
| Peru/KJ627417/15-Jul-2010 | KJ627417 | 15-Jul-10 |
| Peru/KJ627379/31-Dec-2009 | KJ627379 | 31-Dec-09 |
| Peru/KJ627401/10-Feb-2011 | KJ627401 | 10-Feb-11 |
| Peru/KJ627382/18-Jan-2011 | KJ627382 | 18-Jan-11 |
| Canada/KF178981/14-Jan-2009 | KF178981 | 14-Jan-09 |
| India/KC731526/13-Jan-2011 | KC731526 | 13-Jan-11 |
| China/JQ689399/18-Dec-2008 | JQ689399 | 18-Dec-08 |
| India/KC731500/08-Nov-2009 | KC731500 | 8-Nov-09 |
| Canada/KF178976/29-Jan-2009 | KF178976 | 29-Jan-09 |
| China/JQ689394/21-Apr-2008 | JQ689394 | 21-Apr-08 |
| India/KC731524/11-Jan-2011 | KC731524 | 11-Jan-11 |
| Canada/KF178998/23-Mar-2010 | KF178998 | 23-Mar-10 |
| Canada/KF178996/23-Mar-2010 | KF178996 | 23-Mar-10 |
| Canada/KF178989/16-Mar-2010 | KF178989 | 16-Mar-10 |
| Canada/KF178995/05-Mar-2010 | KF178995 | 5-Mar-10 |
| Canada/KF178971/08-Mar-2008 | KF178971 | 8-Mar-08 |
| Canada/KF178973/24-Mar-2008 | KF178973 | 24-Mar-08 |
| Canada/KF178975/17-Jan-2009 | KF178975 | 17-Jan-09 |
| Peru/KJ627410/11-Jul-2009 | KJ627410 | 11-Jul-09 |
| Peru/KJ627424/28-Jan-2009 | KJ627424 | 28-Jan-09 |
| Peru/KJ627426/13-Dec-2008 | KJ627426 | 13-Dec-08 |
| Peru/KJ627429/07-Feb-2011 | KJ627429 | 7-Feb-11 |
| Peru/KJ627406/24-Feb-2010 | KJ627406 | 24-Feb-10 |
| Canada/KF178987/08-Mar-2010 | KF178987 | 8-Mar-10 |
| Peru/KJ627384/08-Feb-2010 | KJ627384 | 8-Feb-10 |
| Peru/KJ627437/21-Jul-2011 | KJ627437 | 21-Jul-11 |
| Peru/KJ627381/15-Jun-2010 | KJ627381 | 15-Jun-10 |
| Peru/KJ627396/28-May-2010 | KJ627396 | 28-May-10 |
| Canada/KF178992/09-Feb-2010 | KF178992 | 9-Feb-10 |
| Canada/KF178983/31-Jan-2010 | KF178983 | 31-Jan-10 |
| Canada/KF178984/28-Feb-2010 | KF178984 | 28-Feb-10 |
| Peru/KJ627430/21-Dec-2009 | KJ627430 | 21-Dec-09 |
| Peru/KJ627385/21-Nov-2010 | KJ627385 | 21-Nov-10 |
| Peru/KJ627402/06-May-2009 | KJ627402 | 6-May-09 |
| Canada/KF178974/29-Feb-2008 | KF178974 | 29-Feb-08 |
| Peru/KJ627392/15-Oct-2008 | KJ627392 | 15-Oct-08 |
| Canada/KF178986/09-Mar-2010 | KF178986 | 9-Mar-10 |
| China/JQ689393/14-Apr-2008 | JQ689393 | 14-Apr-08 |
| China/JQ689396/11-Sep-2008 | JQ689396 | 11-Sep-08 |
| China/JQ689395/04-Sep-2008 | JQ689395 | 4-Sep-08 |
| China/JQ689398/10-Nov-2008 | JQ689398 | 10-Nov-08 |
| China/JQ689397/03-Nov-2008 | JQ689397 | 3-Nov-08 |
| China/GQ153651/15-Dec-2008 | GQ153651 | 15-Dec-08 |

Table S3: GenBank accession numbers for F gene HMPV sequences from Kilifi, Kenya generated in this study

| **Sample/I.D** | **Accession number** | Date |
| --- | --- | --- |
| Kenya/005/14-Jun-07 | KT191355 | 14-Jun-07 |
| Kenya/006/3-Jul-07 | KT191465 | 3-Jul-07 |
| Kenya/010/4-Dec-07 | KT191402 | 4-Dec-07 |
| Kenya/007/11-Oct-07 | KT191436 | 11-Oct-07 |
| Kenya/008/5-Nov-07 | KT191363 | 5-Nov-07 |
| Kenya/009/19-Nov-07 | KT191403 | 19-Nov-07 |
| Kenya/011/12-Dec-07 | KT191397 | 12-Dec-07 |
| Kenya/012/12-Dec-07 | KT191374 | 12-Dec-07 |
| Kenya/013/14-Dec-07 | KT191466 | 14-Dec-07 |
| Kenya/014/20-Dec-07 | KT191377 | 20-Dec-07 |
| Kenya/015/4-Jan-08 | KT191401 | 4-Jan-08 |
| Kenya/016/J8-an-08 | KT191426 | 8-Jan-08 |
| Kenya/017/12-Jan-08 | KT191438 | 12-Jan-08 |
| Kenya/018/15-Jan-08 | KT191427 | 15-Jan-08 |
| Kenya/019/18-Jan-08 | KT191390 | 18-Jan-08 |
| Kenya/020/26-Jan-08 | KT191428 | 26-Jan-08 |
| Kenya/021/29-Jan-08 | KT191429 | 29-Jan-08 |
| Kenya/022/3-Feb-08 | KT191464 | 3-Feb-08 |
| Kenya/023/4-Feb-08 | KT191437 | 4-Feb-08 |
| Kenya/024/13-Feb-08 | KT191391 | 13-Feb-08 |
| Kenya/025/15-Feb-08 | KT191386 | 15-Feb-08 |
| Kenya/027/26-Feb-08 | KT191392 | 26-Feb-08 |
| Kenya/026/26-Feb-08 | KT191479 | 26-Feb-08 |
| Kenya/028/23-Mar-08 | KT191393 | 23-Mar-08 |
| Kenya/029/31-Mar-08 | KT191400 | 31-Mar-08 |
| Kenya/030/4-Apr-08 | KT191388 | 4-Apr-08 |
| Kenya/031/5-Apr-08 | KT191430 | 5-Apr-08 |
| Kenya/032/18-Apr-08 | KT191431 | 18-Apr-08 |
| Kenya/033/12-Jul-08 | KT191404 | 12-Jul-08 |
| Kenya/034/J21-ul-08 | KT191360 | 21-Jul-08 |
| Kenya/035/28-Jul-08 | KT191361 | 28-Jul-08 |
| Kenya/037/23-Aug-08 | KT191362 | 23-Aug-08 |
| Kenya/038/27-Aug-08 | KT191381 | 27-Aug-08 |
| Kenya/039/31-Aug-08 | KT191432 | 31-Aug-08 |
| Kenya/036/3-Jul-08 | KT191472 | 3-Oct-08 |
| Kenya/041/8-Oct-08 | KT191398 | 8-Oct-08 |
| Kenya/042/13-Oct-08 | KT191433 | 13-Oct-08 |
| Kenya/043/21-Oct-08 | KT191414 | 21-Oct-08 |
| Kenya/044/5-Nov-08 | KT191394 | 5-Nov-08 |
| Kenya/045/5-Nov-08 | KT191434 | 5-Nov-08 |
| Kenya/046/8-Nov-08 | KT191444 | 8-Nov-08 |
| Kenya/040/15-Nov-08 | KT191399 | 15-Nov-08 |
| Kenya/047/17-Nov-08 | KT191469 | 17-Nov-08 |
| Kenya/048/19-Nov-08 | KT191395 | 19-Nov-08 |
| Kenya/049/2-Dec-08 | KT191396 | 2-Dec-08 |
| Kenya/050/26-Dec-08 | KT191468 | 26-Dec-08 |
| Kenya/051/30-Dec-08 | KT191480 | 30-Dec-08 |
| Kenya/052/25-Jan-09 | KT191473 | 25-Jan-09 |
| Kenya/053/29-Jan-09 | KT191435 | 29-Jan-09 |
| Kenya/054/9-Feb-09 | KT191364 | 9-Feb-09 |
| Kenya/055/27-Feb-09 | KT191365 | 27-Feb-09 |
| Kenya/056/28-Feb-09 | KT191366 | 28-Feb-09 |
| Kenya/057/4-Nov-09 | KT191367 | 4-Nov-09 |
| Kenya/058/8-Nov-09 | KT191470 | 8-Nov-09 |
| Kenya/059/16-Nov-09 | KT191461 | 16-Nov-09 |
| Kenya/060/16-Nov-09 | KT191446 | 16-Nov-09 |
| Kenya/06217-/Nov-09 | KT191456 | 17-Nov-09 |
| Kenya/061/17-Nov-09 | KT191447 | 17-Nov-09 |
| Kenya/063/30-Nov-09 | KT191368 | 30-Nov-09 |
| Kenya/064/2-Dec-09 | KT191441 | 2-Dec-09 |
| Kenya/065/3-Dec-09 | KT191369 | 3-Dec-09 |
| Kenya/066/3-Dec-09 | KT191385 | 3-Dec-09 |
| Kenya/067/7-Dec-09 | KT191370 | 7-Dec-09 |
| Kenya/068/8-Dec-09 | KT191448 | 8-Dec-09 |
| Kenya/069/9-Dec-09 | KT191482 | 9-Dec-09 |
| Kenya/070/11-Dec-09 | KT191449 | 11-Dec-09 |
| Kenya/071/13-Dec-09 | KT191419 | 13-Dec-09 |
| Kenya/072/13-Dec-09 | KT191450 | 13-Dec-09 |
| Kenya/073/14-Dec-09 | KT191420 | 14-Dec-09 |
| Kenya/074/14-Dec-09 | KT191371 | 14-Dec-09 |
| Kenya/075/15-Dec-09 | KT191421 | 15-Dec-09 |
| Kenya/076/16-Dec-09 | KT191422 | 16-Dec-09 |
| Kenya/077/16-Dec-09 | KT191451 | 16-Dec-09 |
| Kenya/078/18-Dec-09 | KT191372 | 18-Dec-09 |
| Kenya/079/22-Dec-09 | KT191442 | 22-Dec-09 |
| Kenya/080/22-Dec-09 | KT191415 | 22-Dec-09 |
| Kenya/081/26-Dec-09 | KT191471 | 26-Dec-09 |
| Kenya/082/28-Dec-09 | KT191452 | 28-Dec-09 |
| Kenya/083/28-Dec-09 | KT191373 | 28-Dec-09 |
| Kenya/084/29-Dec-09 | KT191375 | 29-Dec-09 |
| Kenya/085/1-Jan-10 | KT191411 | 1-Jan-10 |
| Kenya/086/5-Jan-10 | KT191453 | 5-Jan-10 |
| Kenya/087/12-Jan-10 | KT191454 | 12-Jan-10 |
| Kenya/088/15-Jan-10 | KT191467 | 15-Jan-10 |
| Kenya/089/19-Jan-10 | KT191455 | 19-Jan-10 |
| Kenya/090/20-Jan-10 | KT191443 | 20-Jan-10 |
| Kenya/091/21-Jan-10 | KT191445 | 21-Jan-10 |
| Kenya/092/24-Jan-10 | KT191423 | 24-Jan-10 |
| Kenya/093/25-Jan-10 | KT191481 | 25-Jan-10 |
| Kenya/094/25-Jan-10 | KT191387 | 25-Jan-10 |
| Kenya/095/27-Jan-10 | KT191457 | 27-Jan-10 |
| Kenya/096/1-Mar-10 | KT191424 | 1-Mar-10 |
| Kenya/097/18-Oct-10 | KT191376 | 18-Oct-10 |
| Kenya/098/19-Oct-10 | KT191409 | 19-Oct-10 |
| Kenya/099/19-Oct-10 | KT191410 | 19-Oct-10 |
| Kenya/100/19-Nov-10 | KT191425 | 19-Nov-10 |
| Kenya/101/21-Nov-10 | KT191408 | 21-Nov-10 |
| Kenya/102/23-Nov-10 | KT191384 | 23-Nov-10 |
| Kenya/103/7-Dec-10 | KT191458 | 7-Dec-10 |
| Kenya/104/9-Dec-10 | KT191382 | 9-Dec-10 |
| Kenya/105/13-Dec-10 | KT191356 | 13-Dec-10 |
| Kenya/106/21-Dec-10 | KT191406 | 21-Dec-10 |
| Kenya/107/23-Dec-10 | KT191412 | 23-Dec-10 |
| Kenya/108/11-Jan-11 | KT191383 | 11-Jan-11 |
| Kenya/109/16-Feb-11 | KT191407 | 16-Feb-11 |
| Kenya/110/1-Mar-11 | KT191439 | 1-Mar-11 |
| Kenya/111/2-Mar-11 | KT191484 | 2-Mar-11 |
| Kenya/112/12-Mar-11 | KT191459 | 12-Mar-11 |
| Kenya/113/25-Mar-11 | KT191378 | 25-Mar-11 |
| Kenya/114/4-Apr-11 | KT191357 | 4-Apr-11 |
| Kenya/115/15-Apr-11 | KT191416 | 15-Apr-11 |
| Kenya/116/30-Jun-11 | KT191417 | 30-Jun-11 |
| Kenya/117/12-Jul-11 | KT191440 | 12-Jul-11 |
| Kenya/118/25-Sept-11 | KT191474 | 25-Sep-11 |
| Kenya/119/7-Oct-11 | KT191475 | 7-Oct-11 |
| Kenya/120/13-Nov-11 | KT191413 | 13-Nov-11 |
| Kenya/121/14-Nov-11 | KT191358 | 14-Nov-11 |
| Kenya/122/17-Nov-11 | KT191462 | 17-Nov-11 |
| Kenya/123/24-Nov-11 | KT191418 | 24-Nov-11 |
| Kenya/124/30-Nov-11 | KT191476 | 30-Nov-11 |
| Kenya/125/1-Dec-11 | KT191477 | 1-Dec-11 |
| Kenya/126/5-Dec-11 | KT191389 | 5-Dec-11 |
| Kenya/128/21-Dec-11 | KT191460 | 21-Dec-11 |
| Kenya/129/5-Jan-12 | KT191379 | 5-Jan-12 |
| Kenya/130/16-Jan-12 | KT191463 | 16-Jan-12 |
| Kenya/131/3-Feb-12 | KT191478 | 3-Feb-12 |
| Kenya/132/4-Feb-12 | KT191405 | 4-Feb-12 |
| Kenya/133/8-Feb-12 | KT191483 | 8-Feb-12 |
| Kenya/134/9-Feb-12 | KT191380 | 9-Feb-12 |
| Kenya/135/14-Feb-12 | KT191359 | 14-Feb-12 |

Table S4: GenBank accession numbers for G gene HMPV sequences from Kilifi generated in this study

| **Sample I.D** | **Accession number** | **Date** |
| --- | --- | --- |
| Kenya/146/4-Dec-07 | KT191299 | 4-Dec-07 |
| Kenya/145/18-Dec-07 | KT191300 | 18-Dec-07 |
| Kenya/016/8-Jan-08 | KT191301 | 8-Jan-08 |
| Kenya/018/15-Jan-08 | KT191302 | 15-Jan-08 |
| Kenya/020/26-Jan-08 | KT191303 | 26-Jan-08 |
| Kenya/032/18-Apr-08 | KT191304 | 18-Apr-08 |
| Kenya/036/3-Jul-08 | KT191305 | 3-Oct-08 |
| Kenya/038/27-Aug-08 | KT191306 | 27-Aug-08 |
| Kenya/039/31-Aug-08 | KT191307 | 31-Aug-08 |
| Kenya/041/8-Oct-08 | KT191308 | 8-Oct-08 |
| Kenya/042/13-Oct-08 | KT191309 | 13-Oct-08 |
| Kenya/046/8-Nov-08 | KT191310 | 8-Nov-08 |
| Kenya/048/19-Nov-08 | KT191311 | 19-Nov-08 |
| Kenya/054/9-Feb-09 | KT191312 | 9-Feb-09 |
| Kenya/153/24-Apr-09 | KT191313 | 24-Apr-09 |
| Kenya/150/4-Jun-09 | KT191314 | 4-Jun-09 |
| Kenya/152/17-Jul-09 | KT191315 | 17-Jul-09 |
| Kenya/058/8-Nov-09 | KT191316 | 8-Nov-09 |
| Kenya/059/16-Nov-09 | KT191317 | 16-Nov-09 |
| Kenya/062/17-Nov-09 | KT191318 | 17-Nov-09 |
| Kenya/155/1-Dec-09 | KT191319 | 1-Dec-09 |
| Kenya/064/2-Dec-09 | KT191320 | 2-Dec-09 |
| Kenya/142/5-Dec-09 | KT191321 | 5-Dec-09 |
| Kenya/070/11-Dec-09 | KT191322 | 11-Dec-09 |
| Kenya/071/13-Dec-09 | KT191323 | 13-Dec-09 |
| Kenya/072/13-Dec-09 | KT191324 | 13-Dec-09 |
| Kenya/076/16-Dec-09 | KT191325 | 16-Dec-09 |
| Kenya/081/26-Dec-09 | KT191326 | 26-Dec-09 |
| Kenya/085/1-Jan-10 | KT191327 | 1-Jan-10 |
| Kenya/143/4-Jan-10 | KT191328 | 4-Jan-10 |
| Kenya/141/7-Jan-10 | KT191329 | 7-Jan-10 |
| Kenya/087/12-Jan-10 | KT191330 | 12-Jan-10 |
| Kenya/091/21-Jan-10 | KT191331 | 21-Jan-10 |
| Kenya/094/25-Jan-10 | KT191332 | 25-Jan-10 |
| Kenya/095/27-Jan-10 | KT191333 | 27-Jan-10 |
| Kenya/097/18-Oct-10 | KT191334 | 18-Oct-10 |
| Kenya/100/19-Nov-10 | KT191335 | 19-Nov-10 |
| Kenya/105/13-Dec-10 | KT191336 | 13-Dec-10 |
| Kenya/109/16-Feb-11 | KT191337 | 16-Feb-11 |
| Kenya/148/1-Mar-11 | KT191338 | 1-Mar-11 |
| Kenya/112/12-Mar-11 | KT191339 | 12-Mar-11 |
| Kenya/144/19-Mar-11 | KT191340 | 19-Mar-11 |
| Kenya/147/4-Apr-11 | KT191341 | 4-Apr-11 |
| Kenya/115/15-Apr-11 | KT191342 | 15-Apr-11 |
| Kenya/117/12-Jul-11 | KT191343 | 12-Jul-11 |
| Kenya/158/25-Jul-11 | KT191344 | 25-Jul-11 |
| Kenya/118/25-Sept-11 | KT191345 | 25-Sep-11 |
| Kenya/149/8-Nov-11 | KT191346 | 8-Nov-11 |
| Kenya/154/12-Nov-11 | KT191347 | 12-Nov-11 |
| Kenya/121/14-Nov-11 | KT191348 | 14-Nov-11 |
| Kenya/123/24-Nov-11 | KT191349 | 24-Nov-11 |
| Kenya/124/30-Nov-11 | KT191350 | 30-Nov-11 |
| Kenya/157/7-Dec-11 | KT191351 | 7-Dec-11 |
| Kenya/127/13-Dec-11 | KT191352 | 13-Dec-11 |
| Kenya/128/21-Dec-11 | KT191353 | 21-Dec-11 |
| Kenya/156/21-Dec-11 | KT191354 | 21-Dec-11 |
