# Supplementary figures and images for: Human metapneumovirus epidemiological and evolutionary patterns in Coastal Kenya, 2007-11
Source: BMC Infect Dis. 2016 Jun 17;16:301. doi: 10.1186/s12879-016-1605-0 (PMC4912817; doi:10.1186/s12879-016-1605-0)

## Slide 1
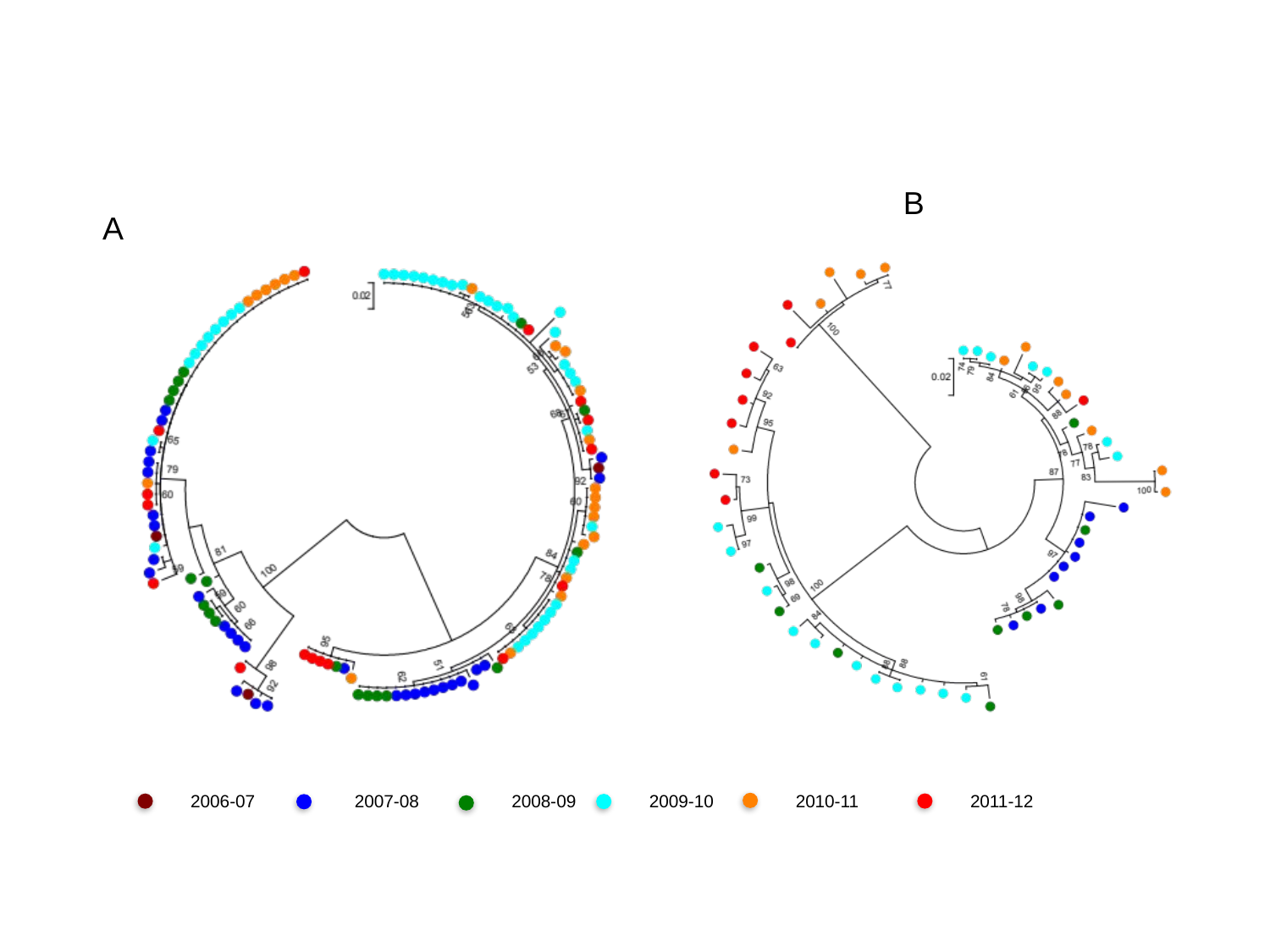

B
A
2006-07
2007-08
2008-09
2009-10
2010-11
2011-12

Supplement: Additional file 2: Figure S1. — ML phylogeny of F and G gene sequences colored by epidemic, from Kilifi 2007-11. Panel A. Using 123 F gene sequences. Panel B. Using 56 G gene sequences. All viruses in Panel B are subgroup A2. (PPTX 175 kb) [file 12879_2016_1605_MOESM2_ESM.pptx]

## Slide 1
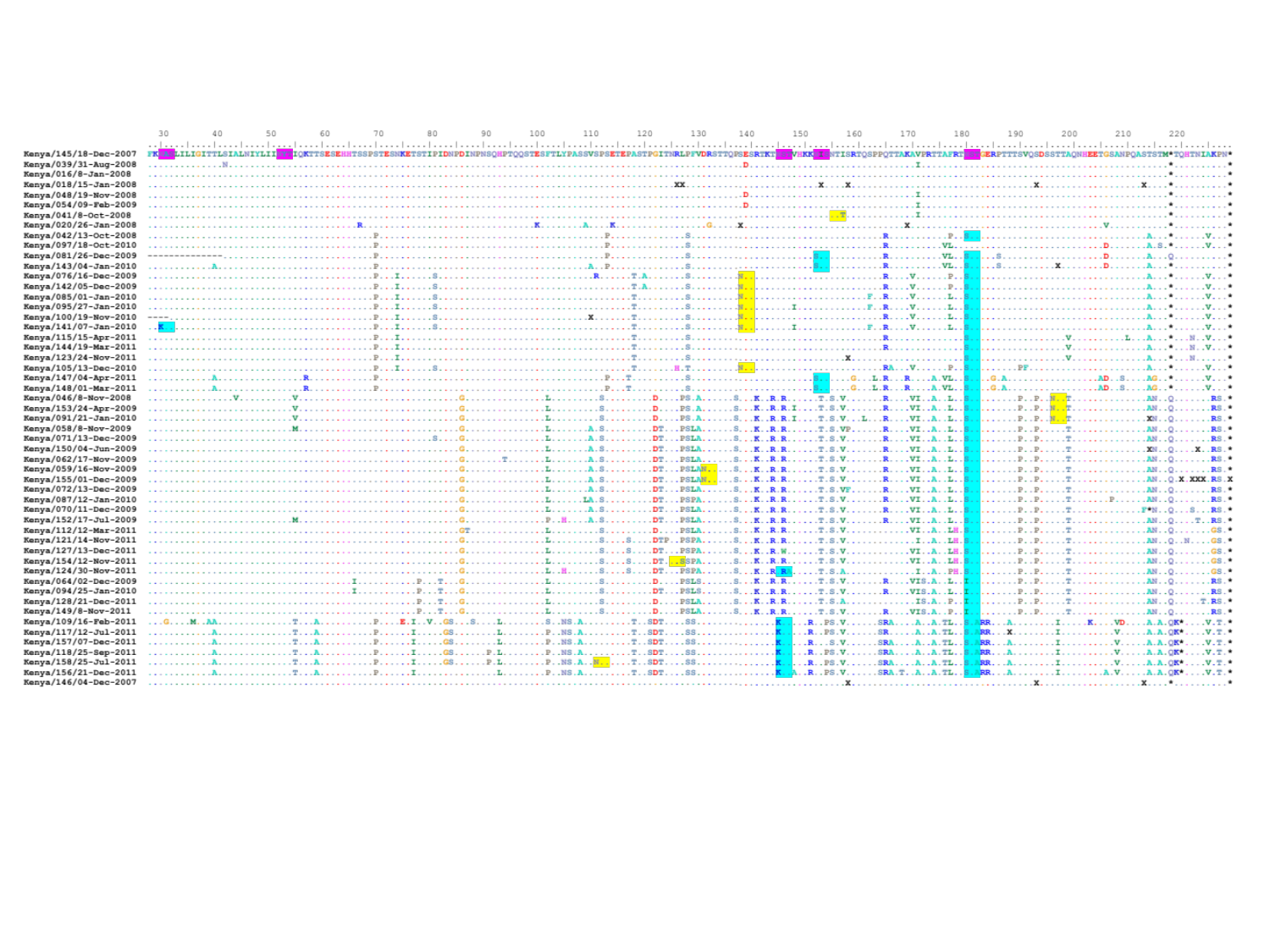

Supplement: Additional file 4: Figure S3. — An amino acid sequence alignment predicted from the 53 unique G gene nucleotide sequences from Kilifi, Coastal Kenya, 2007-11. N-glycosylation sites have been highlighted: pink colour for the identified sites; blue colour for loss and yellow for gains of N-glycosylation sites. (PPTX 445 kb) [file 12879_2016_1605_MOESM4_ESM.pptx]

## Slide 1
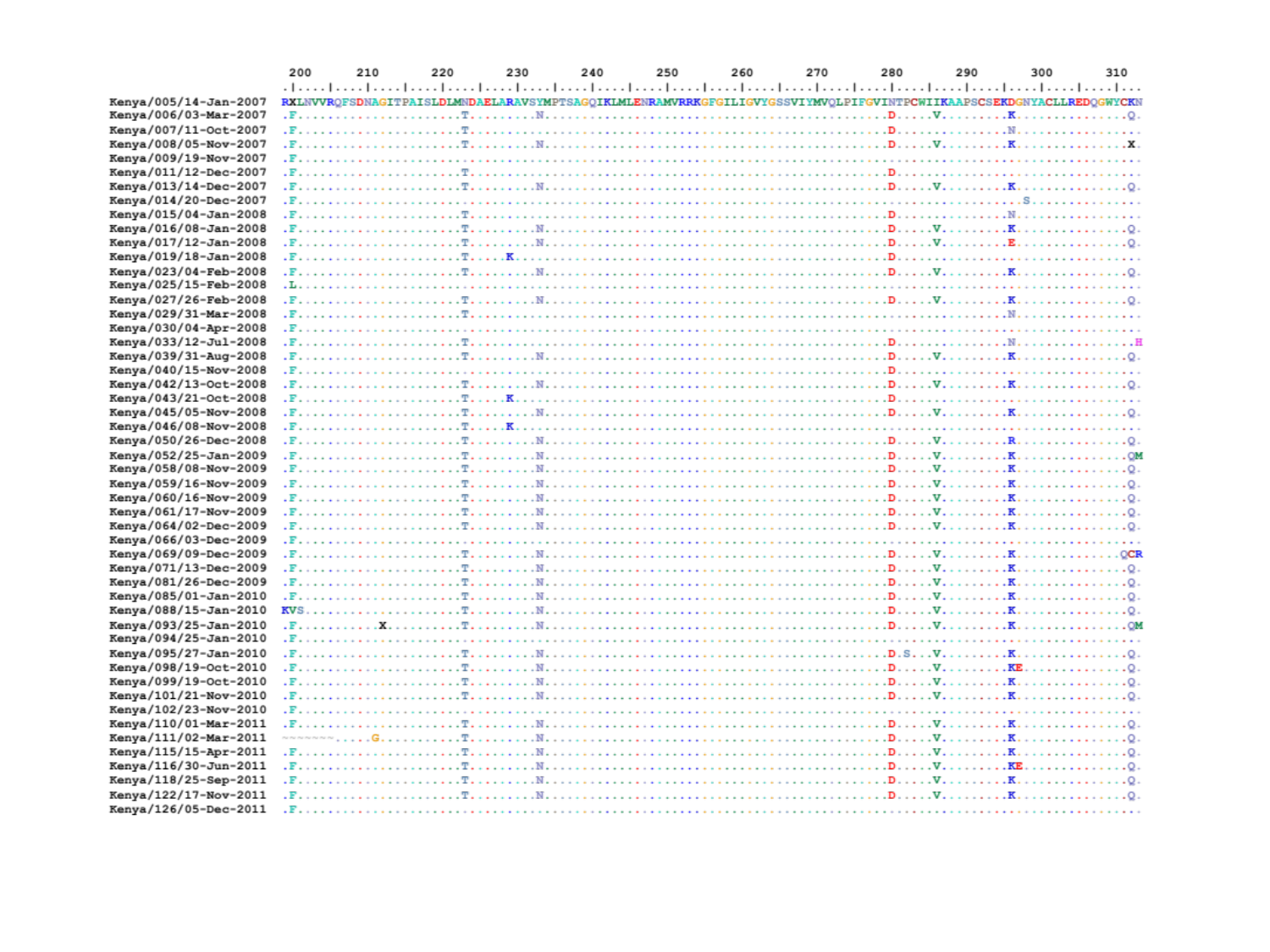

Supplement: Additional file 5: Figure S4. — An amino acid sequence alignment predicted from 49 unique F gene nucleotide sequences from Kilifi, Coastal Kenya, 2007-11. (PPTX 259 kb) [file 12879_2016_1605_MOESM5_ESM.pptx]
