# Supplementary material for: Human metapneumovirus epidemiological and evolutionary patterns in Coastal Kenya, 2007-11
Source: BMC Infect Dis. 2016 Jun 17;16:301. doi: 10.1186/s12879-016-1605-0 (PMC4912817; doi:10.1186/s12879-016-1605-0)
Supplement: Additional file 3: Figure S2. — A. Temporal distribution of HMPV subgroups by month isolated from Kilifi County Hospital in the 5 years of the study (2007-11). Numbers at the bottom indicate number of genotypes each year determined by a combined F and G gene sequencing (total of 123 out of the 160 positives) in Kilifi, Kenya in the period 2007–2011. (PPTX 86 kb) [file 12879_2016_1605_MOESM3_ESM.pptx]

## Slide 1
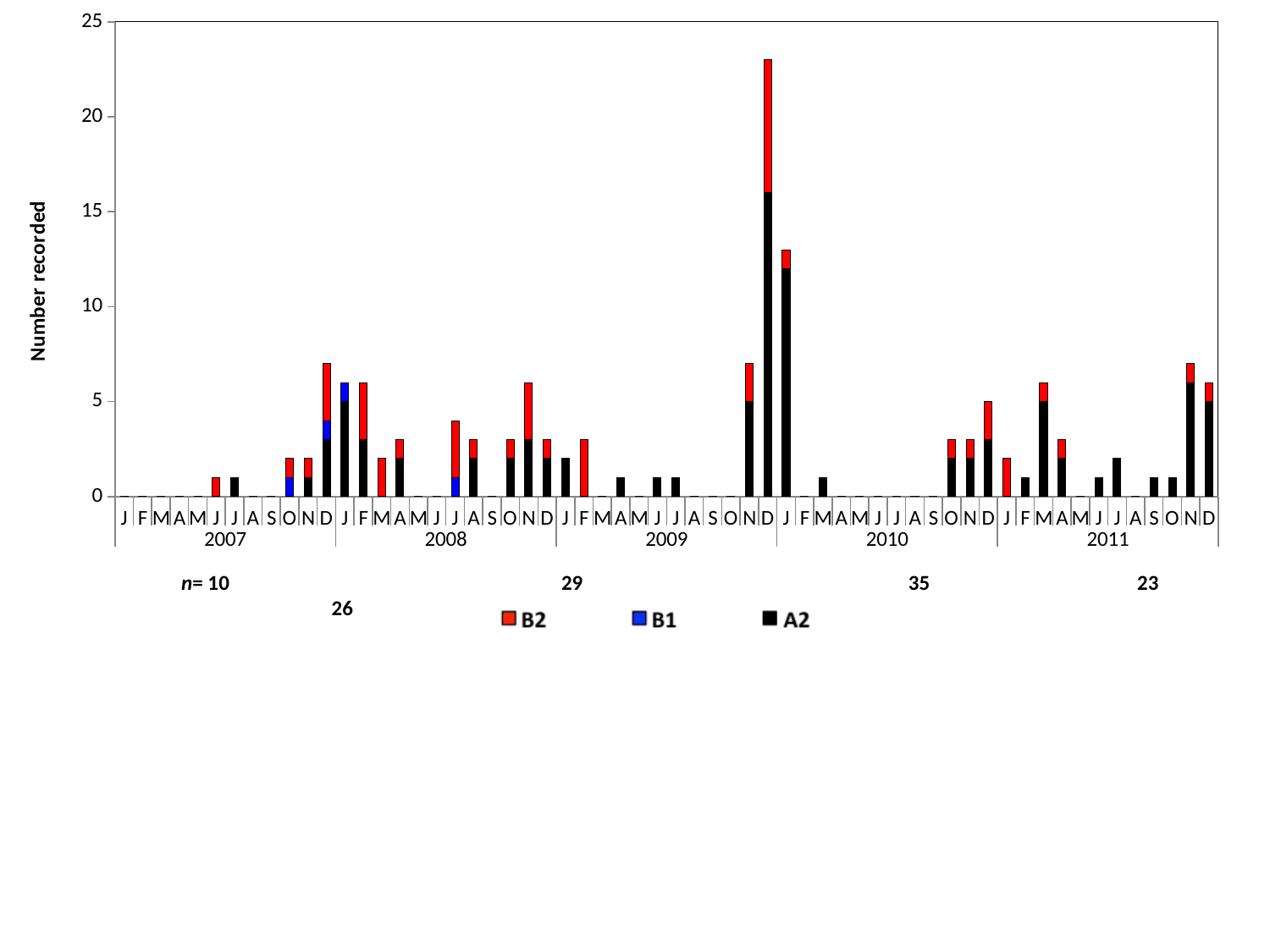

### Chart
| Category | A2b | B1 | B2 |
|---|---|---|---|
| J | 0.0 | 0.0 | 0.0 |
| F | 0.0 | 0.0 | 0.0 |
| M | 0.0 | 0.0 | 0.0 |
| A | 0.0 | 0.0 | 0.0 |
| M | 0.0 | 0.0 | 0.0 |
| J | 0.0 | 0.0 | 1.0 |
| J | 1.0 | 0.0 | 0.0 |
| A | 0.0 | 0.0 | 0.0 |
| S | 0.0 | 0.0 | 0.0 |
| O | 0.0 | 1.0 | 1.0 |
| N | 1.0 | 0.0 | 1.0 |
| D | 3.0 | 1.0 | 3.0 |
| J | 5.0 | 1.0 | 0.0 |
| F | 3.0 | 0.0 | 3.0 |
| M | 0.0 | 0.0 | 2.0 |
| A | 2.0 | 0.0 | 1.0 |
| M | 0.0 | 0.0 | 0.0 |
| J | 0.0 | 0.0 | 0.0 |
| J | 0.0 | 1.0 | 3.0 |
| A | 2.0 | 0.0 | 1.0 |
| S | 0.0 | 0.0 | 0.0 |
| O | 2.0 | 0.0 | 1.0 |
| N | 3.0 | 0.0 | 3.0 |
| D | 2.0 | 0.0 | 1.0 |
| J | 2.0 | 0.0 | 0.0 |
| F | 0.0 | 0.0 | 3.0 |
| M | 0.0 | 0.0 | 0.0 |
| A | 1.0 | 0.0 | 0.0 |
| M | 0.0 | 0.0 | 0.0 |
| J | 1.0 | 0.0 | 0.0 |
| J | 1.0 | 0.0 | 0.0 |
| A | 0.0 | 0.0 | 0.0 |
| S | 0.0 | 0.0 | 0.0 |
| O | 0.0 | 0.0 | 0.0 |
| N | 5.0 | 0.0 | 2.0 |
| D | 16.0 | 0.0 | 7.0 |
| J | 12.0 | 0.0 | 1.0 |
| F | 0.0 | 0.0 | 0.0 |
| M | 1.0 | 0.0 | 0.0 |
| A | 0.0 | 0.0 | 0.0 |
| M | 0.0 | 0.0 | 0.0 |
| J | 0.0 | 0.0 | 0.0 |
| J | 0.0 | 0.0 | 0.0 |
| A | 0.0 | 0.0 | 0.0 |
| S | 0.0 | 0.0 | 0.0 |
| O | 2.0 | 0.0 | 1.0 |
| N | 2.0 | 0.0 | 1.0 |
| D | 3.0 | 0.0 | 2.0 |
| J | 0.0 | 0.0 | 2.0 |
| F | 1.0 | 0.0 | 0.0 |
| M | 5.0 | 0.0 | 1.0 |
| A | 2.0 | 0.0 | 1.0 |
| M | 0.0 | 0.0 | 0.0 |
| J | 1.0 | 0.0 | 0.0 |
| J | 2.0 | 0.0 | 0.0 |
| A | 0.0 | 0.0 | 0.0 |
| S | 1.0 | 0.0 | 0.0 |
| O | 1.0 | 0.0 | 0.0 |
| N | 6.0 | 0.0 | 1.0 |
| D | 5.0 | 0.0 | 1.0 |n= 10		 29		 35	 23	 26
